# Automatic Coregistration of High-Resolution MALDI-MSI and Raman Imaging Applied to Cardiac Tissue of Fabry Disease Mouse Models

**DOI:** 10.1021/acs.analchem.5c07622

**Published:** 2026-06-04

**Authors:** Johann Dierks, Eike Ulrich Brockmann, Anahi-Paula Arias-Loza, Thomas Bocklitz, Peter Nordbeck, Kristina Lorenz, Elena Tolstik, Sven Heiles

**Affiliations:** † 28371 Leibniz-Institut Für Analytische Wissenschaften—ISAS—e.V., Bunsen-Kirchhoff Straße 11, 44139 Dortmund, Germany; ‡ Comprehensive Heart Failure Center, Department of Nuclear Medicine, 27207 University Hospital Würzburg, Am Schwarzenberg 15, 97078 Würzburg, Germany; § 40096 Leibniz Institute of Photonic Technology e.V., Albert-Einstein-Straße 9, 07745 Jena, Germany; ∥ Institute of Physical Chemistry (IPC), Friedrich Schiller University Jena, Helmholtzweg 4, 07743 Jena, Germany; ⊥ Abbe Center of Photonics (ACP), Albert-Einstein-Straße 6, 07745 Jena, Germany; # Department of Internal Medicine I, University Hospital Würzburg, Building A3/A4, Oberdürrbacher Str. 6, 97080 Würzburg, Germany; ¶ Institute of Pharmacology and Toxicology, Julius-Maximilians-University Würzburg, Versbacher Str. 9, 97078 Würzburg, Germany; ∇ Lipidomics, Faculty of Chemistry, University of Duisburg-Essen, Universitätsstr. 2, 45141 Essen, Germany

## Abstract

Understanding early molecular changes in biological tissues is crucial for diagnosing pathological and genetic diseases and for elucidating their underlying mechanisms. However, localized molecular alterations of low-molecular-weight compounds (<2000 Da) are not inferred from conventional staining or genetic methods. Here, we established a multimodal imaging approach that integrates Raman spectroscopy and atmospheric pressure matrix-assisted laser desorption/ionization mass spectrometry imaging (AP-MALDI-MSI): two complementary, label-free techniques enabling molecular profiling of a broad spectrum of biomolecules from one single tissue section. This method was applied to detect globotriaosylceramide (Gb3) accumulation in the heart tissue of murine models of Fabry disease, including mice deficient in α-galactosidase A (GLA) activity (GLA knockout) and transgenic mice with a GLA knockout and an upregulation of Gb3 synthase. With AP-MALDI-MSI, we were able to discern the heterogeneous expression of Gb3 lipoforms with down to 5 μm pixel size and reveal the significantly increased Gb3 content in mice containing a GLA knockout combined with human Gb3 synthase overexpression compared to GLA knockout and wild-type samples. By employing Raman microscopy with a pixel size of 2 μm, we were able to contextualize the physiological alterations in cardiac tissue by identifying components associated with nuclei, tissue, collagen, and lipids for the same three genotypes. An automated coregistration algorithm aligned Raman and AP-MALDI-MSI data from the same tissue section with a (5.1 ± 1.6) μm precision, enabling overlay at 5 and 2 μm resolutions. The method resolved heterogeneous Gb3 distributions and distinct lipid species in cardiac mouse tissue.

## Graphical abstract

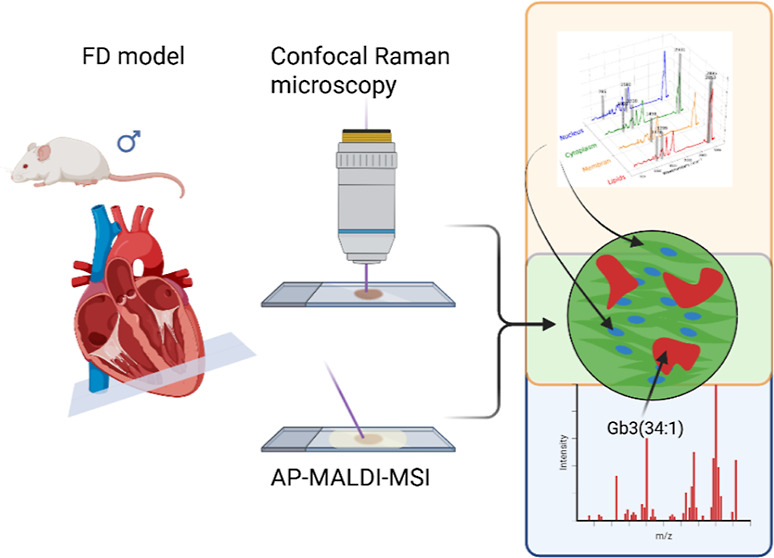

## Introduction

Detection of molecular alterations in tissues can support early diagnosis, elucidate disease pathomechanisms, and facilitate timely implementation of targeted therapeutic options. However, the detection of such alterations in complex biological matrices—often with limited access and availability—remains a significant bioanalytical challenge, as comprehensive molecular profiling of lipid, metabolite, and protein distributions is essential for accurate disease predictions.^1–3^ Even though genetic tests are valuable diagnostic tools for mutation-driven diseases such as cancers or infiltrative cardiomyopathies, they have limited predictive power for disease onset or organ-specific manifestations as they do not capture the local microenvironments that critically influence these processes.^4,5^ Current diagnostic procedures analyzing molecular changes rely on morphological, targeted immunohistochemical or biochemical assays lacking coverage of biomolecules across diverse chemical classes, especially those with low molecular weight (<2000 Da).^6^

Fabry disease (FD) exemplifies these challenges as an X-linked lysosomal storage disorder caused by pathogenic variants in the gene encoding α-galactosidase A (GLA). These variants can result in reduced GLA activity and subsequent accumulation of globotriaosylceramide (Gb3) in diverse cell types.^7^ FD presents as a heterogeneous multisystemic condition ranging from multiorgan involvement to preferential cardiac or neurological involvement, with phenotypes varying significantly due to gender, genotype, age, and unidentified genetic modifiers.^8^ Despite the availability of genetic tests and assays for blood lyso-Gb3 and Gb3 levels,^9^ the individual and heterogeneous deposition patterns of Gb3 make it difficult to infer the specific risk for kidney, brain, or heart damage and to determine optimal therapeutic strategies for individuals.^10^ To address these diagnostic limitations, spatially resolved bioanalytic workflows offering untargeted molecular insights are needed as tools to provide additional information for risk stratification and therapeutic guidance.

Several vibrational spectroscopic methods have proven promising for investigating untargeted local biomolecular changes in healthy versus diseased tissue such as mid-IR spectroscopy or Raman spectroscopy, coherent anti-Stokes Raman scattering (CARS), and stimulated Raman scattering (SRS) spectroscopy.^11,12^ Mid-IR spectroscopy detects molecular vibrations in the mid-infrared range and provides information on the overall chemical composition, particularly sensitive to functional groups that are predominantly arising due to the presence of lipids, proteins, or carbohydrates, and with the spatial resolution typically limited to about 3 μm.^13^ Raman spectroscopy relies on inelastic scattering of visible or near-infrared light, enabling high chemical specificity and spatial resolution down to ∼400 nm, allowing detailed mapping of biochemical components such as lipids or proteins.^14–17^ CARS and SRS are nonlinear Raman-based techniques that enhance signal intensity and imaging speed, enabling label-free visualization of biomolecules in tissues with submicrometer resolution. All these methods allow identification and spatial mapping of various molecular compound groups using molecular fingerprints, providing complementary information, for a more detailed understanding of disease-related biochemical changes.

Previously, Raman imaging was employed to study lipid distributions in various cell types.^18,19^ Further, Hollon et al. used SRS to distinguish tumorous from healthy brain tissue in glioblastoma and guide intraoperative decision-making.^20^ Despite these potential diagnostic applications and their analytical merits, determining the molecular identities of endogenous compounds remains challenging. This is particularly the case for compounds present at low concentrations or with heterogeneous spatial distributions, which complicate biomarker identification.^19^

Mass spectrometry imaging (MSI) methods such as matrix-assisted laser desorption/ionization (MALDI) provide detailed, spatially resolved information for many biomolecules in tissues. Atmospheric pressure (AP-) MALDI-MSI can detect changes in biomolecular profiles or distinct biomolecules, for example, distinguishing between healthy liver and granuloma.^21^ However, despite molecular specificity offered by MSI, the pixel resolution is often limited to 5–25 μm, and certain compound classes such as proteins are technically difficult to detect.^22,23^ Advanced experimental setups like AP-SMALDI^24^ or transmission mode MALDI with laser postionization^25^ and plasma postionization,^26^ respectively, can achieve pixel resolutions of approximately 1–2 μm and therefore pixel sizes without oversampling below 2 μm by using sophisticated laser optics and ion source geometries. However, they are associated with increased complexity in operation and require trade-offs between signal intensity and pixel resolution. Therefore, a bioanalytical workflow that combines molecular compound annotation via MSI with high lateral resolution and compound class specificity via Raman imaging is highly desirable to minimize analytical drawbacks and maximize information obtained from a single tissue section.

Indeed, previous proof-of-concept studies have demonstrated that Raman imaging and MSI provide complementary information when applied to biological tissues. The combination of Raman imaging and AP-MSI resolved histological features of mouse brain at lateral resolutions of 25 and 75 μm, respectively.^27–29^ Desorption electrospray ionization combined with Raman imaging enhanced molecular insights over Raman imaging or MSI alone in a multiple sclerosis mouse model.^30^ Streamlined pipelines such as RaMALDI have extended these approaches to diverse tissues including liver, kidney, and brain.^28^ Additionally, multiple automatic workflows have already been presented, which combine MALDI-MSI with complementary imaging modalities to enable a coregistration of imaging data.^31–35^ These presented studies allow capturing complementary molecular information; however, they do not always allow automatic alignments for selective regions of interest (ROIs). This is often the case if ROIs lack global alignment points. In particular, the coregistration of images of low pixel sizes with recordings of small ROIs is particularly difficult due to the absence of individual ablation marks.

Here, we contribute to the field by combining high-resolution Raman imaging (2 μm spatial resolution) with AP-MALDI-MSI (5 μm spatial resolution) in heart tissue from FD mouse models. Using automated image alignment, we achieved micrometer-level coregistration, enabling mapping of heterogeneous Gb3 accumulation together with nucleic acid, lipids, protein, and collagen distributions from one tissue slice. This approach not only enables higher spatial resolution for both modalities but also provides detailed molecular insights of FD-associated alterations in cardiac tissue, demonstrating the capabilities of hyperspectral multimodal imaging for the investigation of cardiac tissue.

## Materials and Methods

### Solvents and Chemicals

Ammonium acetate (≥97%, 238,074, Sigma-Aldrich), ammonium formate (LiChropurTM, ≥99.0%, 70,221, Supelco), methyl-*tert*-butylether (MTBE, LiChrosolv, 101,845, Supelco), acetone (LiChrosolv, 100,020, Supelco), Mayer’s Hematoxylin (MHS32-1L, Sigma-Aldrich), Eosin G (yellowish, 1.15935.0100), ethanol (EMPLURA, ≥99.5%, 818,760, Supelco), 2-propanol (used for matrix washing, ≥99.8%, 33539 M, Sigma-Aldrich), Eukitt quick-hardening mounting medium (03989), SPLASH Lipidomix mass spectrometry standard (330,707, Avanti Polar Lipids LLC), and C17:0 Gb3 (860699P, Avanti Polar Lipids LLC) were all purchased from Merck KGaA, Darmstadt, Germany. Formic acid (liquid chromatography–mass spectrometry (LC–MS) grade, 98%, 56,302) was bought from Fluka, Honeywell International Inc., Charlotte, NC, USA. Methanol (methanol absolute ULC/MS–CC/SFC, 136,841), water (water ULC/MS–CC/SFC, 232,141), 2-propanol (2-propanol ULC/MS–CC/SFC, 162,641), and acetonitrile (acetonitrile ULC/MS–CC/SFC, 012,041) were procured from Biosolve BV, Valkenswaard, NL. 2,5-Dihydroxyacetophenone (2,5-DHAP, ≥98%, A12185 was purchased from Alfa Aesar, Ward Hill, MA, USA. ROTIHistol (6640.1) was bought from Carl Roth GmbH + Co. KG, Karlsruhe, Germany, and Milli-Q water was produced by an ELGA PURELAB flex 2 system (18.2 MΩ cm, ELGA LabWater Deutschland, Celle, Germany), 4% paraformaldehyde (PFA), Dulbecco’s phosphate-buffered saline (PBS, Gibco, Thermo Scientific, Waltham, USA), and a commercial Gb3 mixture (Ceramide trihexoside, Cat. No. 1067, Matreya, USA).

### Mouse Model

Male mice aged 18–20 weeks of a C57BL/6J genetic background were used for analysis. The genotypes are GLA^WT^ mice, which represent the wild-type (WT) phenotype, hemizygous GLA^KO^ mice, which carry a targeted X-linked deletion of the α-Gal A gene resulting in a complete loss of enzyme activity,^36^ and G3Stg/GLA^KO^ mice, a model that combines the α-Gal A knockout with the expression of human Gb3 synthase.^37^ All animal procedures were conducted in compliance with German animal welfare regulations and approved by the regional authority for animal research (Landesamt für Verbraucherschutz and Ernährung NRW, Germany; approval number Az. 81-02.04.2021. A464).

### Cryosectioning

Heart tissues were sectioned into 20 μm thick sections at −22 °C using a cryotome (Thermo Scientific, Waltham, USA) after attachment of tissues with ice water to the cryotome holder. The resulting sections were placed on CaF_2_ slides of a 2 mm thickness (Korth, Kiel, Germany) or microscopic glass slides (Th. Geyer GmbH & Co. KG, Renningen, Germany). The slides were stored at −80 °C until further use.

### Raman Data Acquisition

On the day before the Raman measurements, samples were moved from the −80 °C freezer to the −20 °C freezer for slow thawing. The sections were fixed at room temperature (RT) in a 4% PFA solution for 10 min, washed with PBS and afterward vacuum-dried (Concentrator plus, Eppendorf SE, Hamburg, Germany) for 15 min. Several red marks were added around the tissue sections for Raman/MALDI coregistration.

Raman images were acquired using a confocal Raman microscope (alpha 300R, WITec, Ulm, Germany). Bright-field (BF) images were acquired for both tissue overview and selected ROIs. For all Raman measurements, a 785 nm excitation laser beam was focused on the sample using a 50*x*/0.75 NA dry objective (Zeiss, Jena, Germany). A laser power of 100 mW was used, and the integration time was set to 2 s. The pixel resolution of all images was 2 μm. A 300 grooves/mm grating was used for data acquisition. ROIs were selected randomly based on the tissue integrity visualized by the BF image. The ROIs were defined with a size between 150 μm × 150 and 200 μm × 200 μm, resulting in a pixel size between 75 × 75 pixels and 100 × 100 pixels. With the mentioned integration time, the acquisition time was between approximately 3 and 5.5 h.

For reference spectra, a commercial Gb3 mixture was dried and measured in single-point measurements using a 785 nm laser source with a 50 mW power, a 30 s integration time, and 10 accumulations.

### Raman Data Analysis

After preprocessing (see Supporting Information), images were analyzed individually using a combination of hierarchical cluster analysis (HCA) and a multicurve resolution with alternating least-squares (MCR-ALS). First, the measurements were clustered individually with the Ward algorithm and Euclidean distance metric. Afterward, cluster partitions with 10 clusters and 100 clusters were selected for investigation and visualization. Subsequently, the mean spectra for the number of clusters equal 100 of all images that were merged into one single data set and MCR-ALS was performed to detect important chemical components (HCA + MCR-ALS). MCR-ALS aims to reconstruct this data analysis matrix *D* as follows

$$D={CS}^{T}+E$$

where *C* denotes the concentration or abundance matrix, *S* ^T^ is the transposed spectral component matrix, and *E* is the residual errors, which are used to describe each pixel spectrum and to create abundance distribution.^38^ MCR-ALS was applied with the pymcr 0.5.1 package^39^ with ordinary least-squares regressors for both the concentration and the components. Constraints were set to nonnegativity for components and nonnegativity plus normalization to 1 for concentrations. The number of components was manually varied, and the minimal number with the appearance of a lipid-associated component was used for final analysis. Spectra were initialized using classical *k*-means clustering with default settings of the scikit-learn package. For statistical analysis, components were tested individually, computing the mean abundance per mouse based on the tissue sections. Normal distribution was tested with the Shapiro–Wilk test, and the Levene test was used for homogeneity testing. If successful, one-way ANOVA with the Bonferroni correction was applied and—if successful—a post hoc Tukey test was used to detect group-specific significances.

### Sample Preparation for AP-MALDI-MSI

For AP-MALDI-MSI sample preparation, sections on glass slides were thawed and dried in a desiccator for 30 min. Matrix was applied to the samples using a custom-built sublimation chamber (ChemGlass). A total of 250 μL of a 20 mg/mL 2,5-DHAP solution in acetone was added to the sublimation chamber, and the acetone was completely evaporated. Vapor deposition was performed at 130 °C for 4 min under vacuum. The sample was cooled with water ice to 0.5 °C. To prevent recrystallization due to humidity, the sample was immediately transferred to a desiccator for 30 min. As quality control measure, BF images of the sample were recorded before matrix application, after matrix application, and after AP-MALDI-MSI acquisition.

### AP-MALDI-MSI Data Acquisition

AP-MALDI-MSI measurements were conducted on a QExactive HF orbital trapping mass spectrometer (Thermo Fisher Scientific, Bremen, Germany) equipped with an AP-MALDI^5^ AF ion source (TransMIT, Gieβen, Germany). Samples were analyzed at a pixel resolution of 25 × 25 μm^2^ (full pixel mode) and 5 × 5 μm^2^ (spot mode).^40^ The global 20% attenuator was activated, and the variable attenuator was set to 38°. Data were acquired in positive-ion mode with a scan range of *m*/*z* 200–2000 for 25 μm measurements and *m*/*z* 500–1500 for 5 μm measurements. The mass resolution was set to 240,000, the automatic gain control was disabled, and the maximum injection time was 500 ms. The acceleration voltage was set to 3.5 kV for 25 μm measurements and 2.5 kV for 5 μm measurements. The capillary temperature was maintained at 275 °C, and the S-lens RF level was set to 100.

### AP-MALDI-MSI Data Analysis

Thermo. RAW-files and TransMIT. udp-files were converted to .imzML format using the RAW + UDP to IMZML Converter (v1.8r3, TransMIT, Gieβen, Germany). The resulting .imzML files were processed using LipostarMSI (v2.1.0b7, Molecular Horizon, Bettona, Italy).^41^ Data were recalibrated based on the lock masses *m*/*z* 826.5723 (PC(36:1), [M + K]^+^) for 25 μm measurements and *m*/*z* 756.5514 (PC(32:0), [M + Na]^+^) for 5 μm measurements. The recalibrated data were exported as .imzML-files and uploaded to Metaspace^42^ (https://metaspace2020.org) for preliminary lipid annotation.

For statistical analysis of Gb3 accumulation in cardiac tissue, five AP-MALDI-MSI data sets for each genotype were loaded into a single LipostarMSI session and normalized to the root-mean-square (RMS) intensity. Hot-spot removal by histogram equalization was used as provided by the Lipostar MSI software. Normalized data were segmented by using bisecting *k*-means clustering and spatial-aware processing. ROIs covering the tissue section were defined on the basis of the previous segmentation. The mean intensities for the molecules of interest were then extracted and processed in Prism (v10.2.3, GraphPad Software, LLC, Boston, MA, USA). For statistical analysis, normal distribution was tested with the Shapiro–Wilk test, and the Grubbs method was used for the identification of potential outliers. Variance analysis was performed using a one-way ANOVA with a Bonferroni correction. For this, we assume that the variance between groups is bigger than the biological variance within a group. Since all Gb3 species in the GLA^WT^ group were below the detection limit, resulting in mean intensities of zero, this group was excluded from statistical analysis due to the lack of normal distribution and variance. Consequently, an unpaired *t*-test was conducted for comparison between the remaining two groups.

The nomenclature for the lipid species follows the LIPID MAPS shorthand notation guidelines (https://www.lipidmaps.org/lipid_nomenclature).^43,44^

### Overlay of AP-MALDI-MSI and Raman Measurements

For the combination of both measurement modalities, an in-house written application in python 3.12 utilizing the opencv (https://opencv.org/) and scikit-image 0.25.2 packages was developed.^45^ First, Raman measurements were inserted at their positions in the Raman BF overview, which included the whole tissue section with red markers based on coordinates retrieved from system metadata. The metadata were automatically delivered by the system after each Raman measurement and included the *x*,*y* position in the coordinate system. Afterward, a pattern matching algorithm between the BF overview image and BF taken in advance of each Raman measurement was applied to optimize the position. Pattern matching was achieved using a fast normalized cross-correlation algorithm (implemented via the match_template function of the scikit-image package) with variation of the *x*, *y* position. The region of interest to detect the optimal position was set to a 1 mm^2^ region around the position determined by the metadata. The position of maximum response was computed and stored as the final coordinate.

In a second step, the AP-MALDI-MSI measurements were localized in the MALDI-associated BF overview (that was recorded after the measurements). Edge detection maps were computed for each BF overview image using a holistically nested edge-detection neural network.^46^ The network was not pretrained on the images and integrated using the dnn function of the opencv package. Images were forward passed through the network, and an edge detection map was used for ongoing computations. Detection of rectangular structures was achieved by mask convolution on the BF overview image. The mask was designed based on the pixel size of the AP-MALDI MS measurement and the ratio between AP-MALDI-MSI resolution and BF overview resolution, where the estimated measurement border was set to 255 with a thickness of 5 pixels. The outer and inner edges were set to 1. The remaining pixels were set to 0, excluding them from computation. The signal of maximum response was computed to determine the optimal position. This process was repeated twice, first just by varying the mask position in the image to determine a general region of interest and afterward varying the mask position and angle in this ROI.

The final step was the coregistration of the BF overview images and consequently all measurements. Red markers were detected in both images by converting the image to HSV color space, just keeping the saturation dimension (which is far higher for artificial marker pens compared to biological tissue) and applying Otsu’s thresholding to extract them. Afterward, morphological operations in the form of hole closing and removing small objects below 10 μm were applied. Then, the affine matrix describing translation, rotation, and scaling was computed between the BF overviews by applying the cv2. estimateAffinePartial function. For Raman imaging and MSI overlays, 5 μm AP-MALDI-MSI pixels were scaled to 2 μm pixels using the nearest-neighbor interpolation to display these two modalities in one singular image. This matrix was stored and used for all coordinate translation.

After coregistration, overlapping regions were determined by coordinate comparison. For merging, the MCR-ALS results of the Raman analysis were overlapped with the AP-MALDI-MSI specific results of Gb3. To determine if each pixel signal per component is signal or background, classical Otsu’s thresholding was applied for each component, and the signal was merged into one single image.

## Results and Discussion

### Study Design

In order to investigate molecular changes during disease development, we used three FD mouse models, including wild-type, GLA^KO^, and G3Stg/GLA^KO^ mice. The wild-type (GLA^WT^) mice served as the control group, GLA^KO^ mice, lacking GLA, as a model for early-stage or asymptomatic FD phenotype with moderate Gb3 accumulations,^36^ and G3Stg/GLA^KO^ mice, GLA^KO^ mice combined with overexpression of human Gb3 synthase in order to further increase Gb3 depositions,^37^ as a model for a symptomatic FD phenotype including renal and cardiac dysfunctions.^37,47^

First, aiming to establish a comprehensive workflow for the implementation of AP-MALDI-MSI and Raman imaging and their simultaneous application, we started with separate measurements on consecutive sections of cardiac tissue (left ventricular mouse heart sections) by Raman imaging and AP-MALDI-MSI. These parallel measurements provided information for the localization of biochemical components in the cardiac tissue along with specific molecular identification and spatial distribution analysis of distinct Gb3 species. Next, we performed sequential Raman imaging and AP-MALDI-MSI from a single tissue section and established comprehensive multimodal overlays of molecular images from both modalities. This overall procedure aims to maximize the combined molecular information, to differentiate between the three genotypes, and to achieve an automated overlay of Raman imaging and AP-MALDI-MSI modalities (Figure 1).

**Figure 1:**
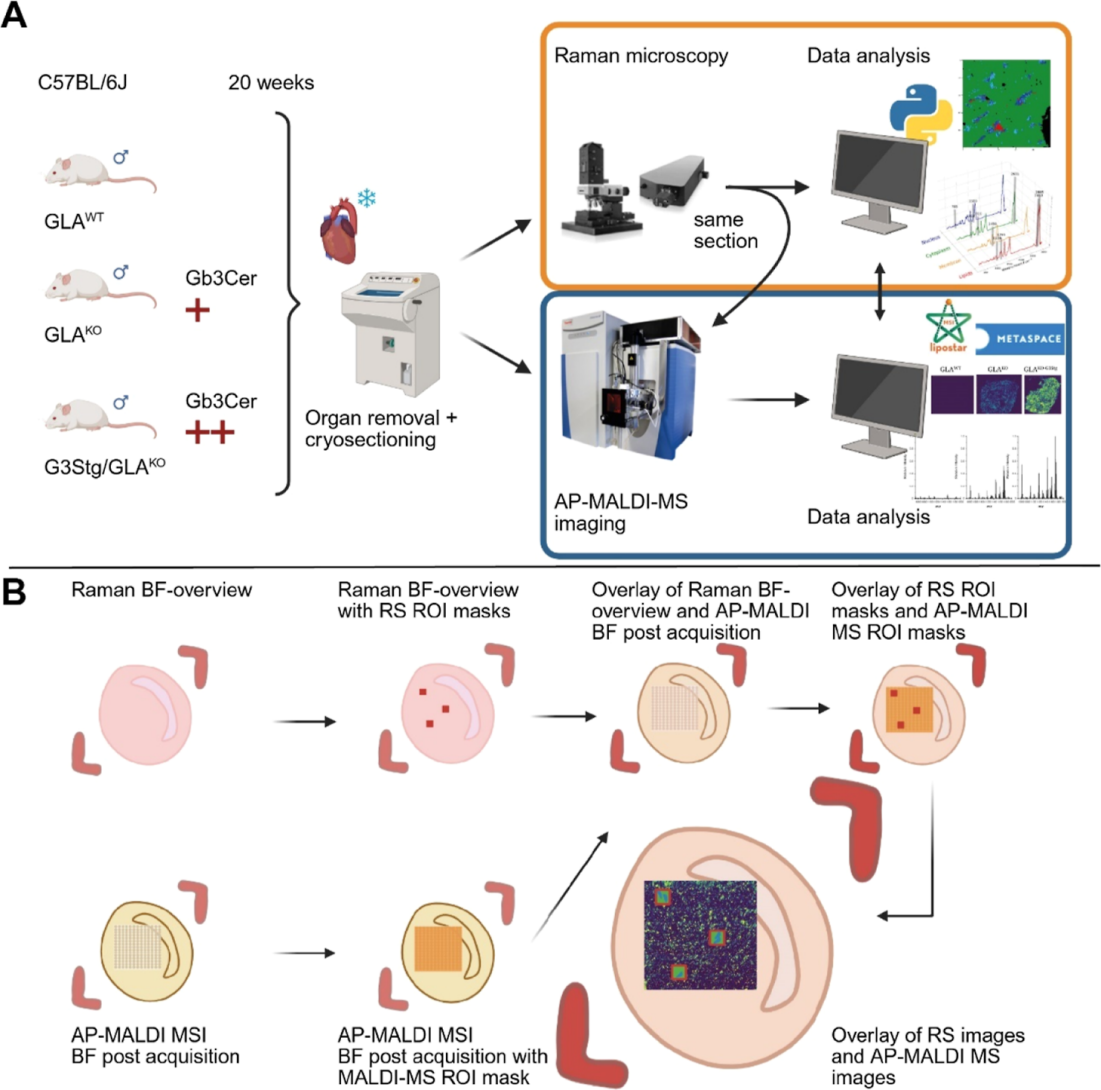
(A) Study concept: Determination of cardiac Gb3 accumulation in an FD mouse model, consisting of wild-type (GLA^WT^), GLA knockout (GLA^KO^), and knockout with additional human Gb3 synthase expression (G3Stg/GLA^KO^) mice, was performed on heart cryosections from 20 week old animals. Initially, AP-MALDI-MSI and Raman microscopy measurements were conducted on consecutive sections. Subsequently, both techniques were applied to the same section, first using Raman imaging, followed by AP-MALDI-MSI, and data analysis. The resulting data sets from both modalities, namely, data of consecutive sections and of sections with combined measurements, were integrated to enhance the depth of information. (B) Schematic presentation of the automated image coregistration of the MALDI-MSI and Raman modalities. Created in https://BioRender.com.

### AP-MALDI-MSI of Gb3 in Murine Cardiac Tissue

First, we aimed to determine the detection capability of AP-MALDI-MSI for Gb3 accumulation in heart tissue across the three experimental groups: GLA^WT^, GLA^KO^, and G3Stg/GLA^KO^ mice. For this purpose, heart tissue sections were scanned with a 25 μm step size and statistical analysis was employed for ROIs covering the entire tissue section of each AP-MALDI-MSI measurement. Figure 2A shows BF images of heart tissue sections alongside the corresponding AP-MALDI-MSI measurements and postacquisition H&E staining, displaying spatial intensity distributions for Gb3 (34:1) (*m*/*z* 1062.635; [M + K]^+^), Gb3 (42:2) (*m*/*z* 1172.744; [M + K]^+^), and PC (34:1) (*m*/*z* 798.542; [M + K]^+^). Figure 2B contains exemplary single pixel mass spectra for each group with the respective Gb3 lipoforms annotated based on the total number of carbon atoms and C=C double bonds in the FA chains. The statistical analysis of ROIs is shown in Figure 2C as violin plots for Gb3 (34:1), Gb3 (42:2), and PC (34:1).

**Figure 2:**
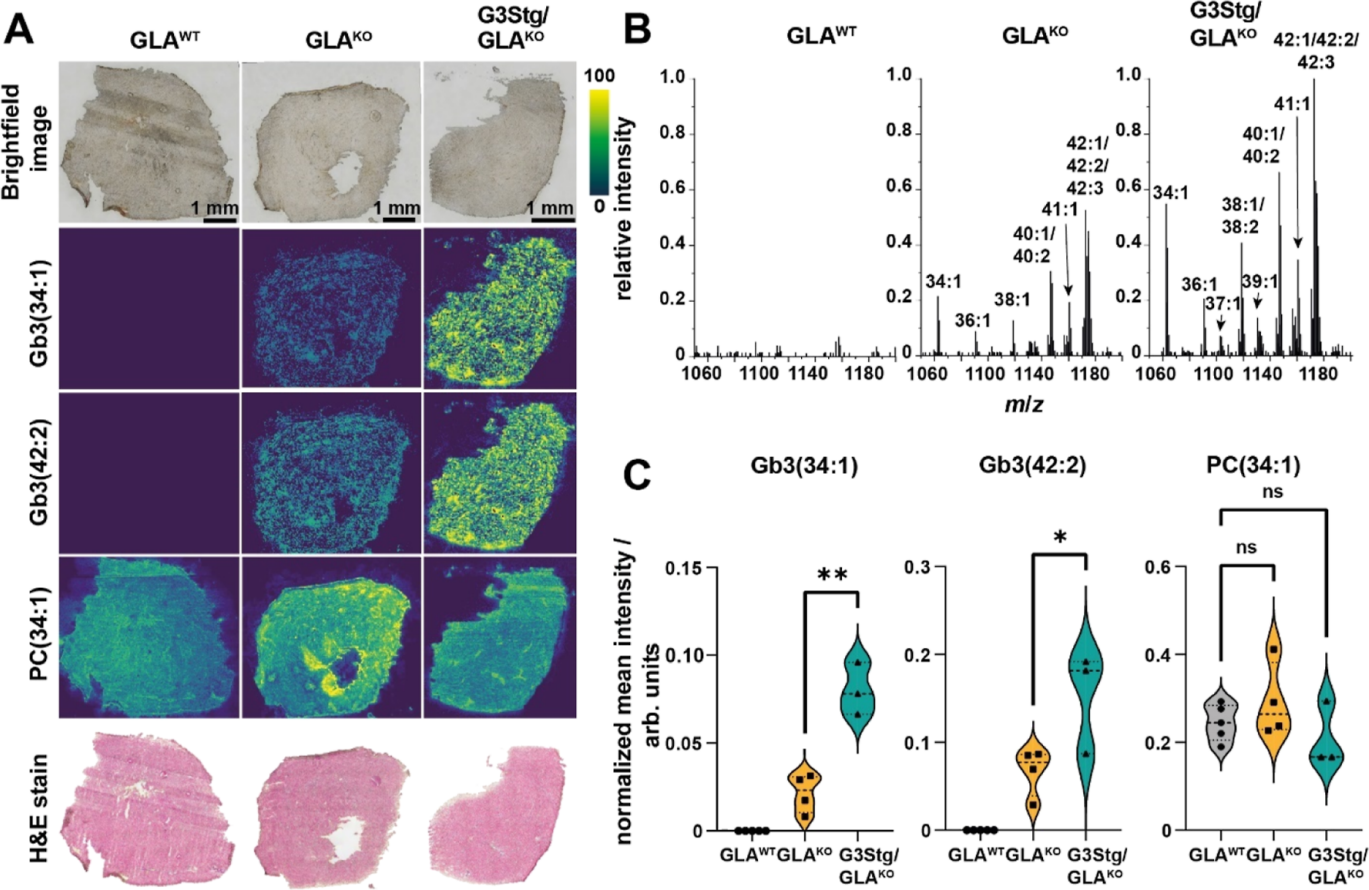
Gb3 accumulation from AP-MALDI-MSI measurements. (A) Visualization of Gb3 accumulation in the murine cardiac tissue of the GLA^KO^, G3Stg/GLA^KO^, and GLA^WT^. Exemplary intensity distributions are presented for Gb3 (34:1) (*m*/*z* 1062.6345; [M + K]^+^), Gb3 (42:2) (*m*/*z* 1172.74380; [M + K]^+^), and PC (34:1) (*m*/*z* 798.5418; [M + K]^+^). Data were acquired with a pixel size of 25 × 25 μm^2^ in full pixel mode and RMS-normalized. Relative intensity values for ion image intensity scale bars are shown in %. (B) Comparison of representative single-pixel mass spectra in the range from *m*/*z* 1050 to 1200. Annotation was performed based on the accurate mass of [M + K]^+^-adducts and MS/MS analysis. (C) Statistical analysis of the Gb3 accumulation was conducted using AP-MALDI-MSI measurements of murine cardiac tissue. The displayed intensities represent mean ROI intensities of the tissue area and were calculated following hot-spot removal by histogram equalization and RMS normalization. GLA^WT^ *n* = 5, GLA^KO^ *n* = 4, G3Stg/GLA^KO^ *n* = 3 biological replicates. **, *p* < 0.01; *; *p* < 0.05; ns = not significant.

Based on the color-coded intensity maps in Figure 2A, a pronounced Gb3 accumulation in the tissue of G3Stg/GLA^KO^ mouse hearts relative to GLA^KO^ samples was measured. Notably, Gb3 signals were absent in the GLA^WT^ control tissue. Additional Gb3 lipoforms identified based on AP-MALDI-MSI and LC–MS/MS analysis are summarized in Figures S1 and S2, Table S1. The ubiquitous lipid PC (34:1) served as an internal quality control exhibiting no statistically significant alterations between genotypes and the identification of tissue outlines as well as the conformation of comparability of MSI data sets.

Using AP-MALDI-MSI, we detected and annotated 12 distinct Gb3 species in G3Stg/GLA^KO^ samples and nine in GLA^KO^ samples, whereas no Gb3 species were detected in GLA^WT^ samples (Figure 2B). Validation of the annotated lipids detected by AP-MALDI-MSI was performed using LC–MS/MS of pooled lipid extracts, which identified 20 Gb3 species based on characteristic MS/MS fragmentation patterns (Table S1). This dual approach confirmed not only the molecular identity of the AP-MALDI-MSI annotations but also their capability to capture the spatial resolution of predominant Gb3 variants in the tissue.

Not only Gb3 (34:1) and Gb3 (42:2) revealed a significantly enhanced accumulation of these disease-specific lipid species in myocardial Fabry samples of the G3Stg/GLA^KO^ genotype compared to GLA^KO^ (Figure 2C) but also other Gb3 compounds were significantly upregulated (Figure S1).

Our data confirm the accumulation of Gb3 in the FD mouse model with elevated Gb3 intensities in the GLA^KO^ samples due to the hindered degradation of Gb3 and even higher intensities in the G3Stg/GLA^KO^ samples, which can be explained by a higher production of Gb3 through the insertion of G3Stg. Through our refined AP-MALDI-MSI method, we visualized the distribution of the most abundant Gb3 lipoforms as described in the literature.^48^ In the GLA^WT^, Gb3 signals were absent or below the limit of detection (LOD) of our AP-MALDI-MSI method. Of note, we could not detect lyso-Gb3 and analogues in the heart tissue of FD mice (GLA^KO^ or G3Stg/GLA^KO^) using AP-MALDI-MSI, consistent with lyso-Gb3 concentrations having been reported about 50× lower than Gb3 concentrations in human heart tissue homogenates.^49,50^

To assess whether the heterogeneous distribution of Gb3 in myocardial tissue is dependent on the Gb3 lipoform and if it correlates with distinct cell types, we conducted high-resolution AP-MALDI-MSI measurements with higher spatial resolution and streamlined the workflow for multimodal imaging.

### Determination of Heterogeneous Gb3 Lipoform Accumulation

Figure 3A shows heterogeneous Gb3 distribution across murine cardiac tissue, with accumulation appearing as localized intensity spots. To improve the resolution of our AP-MALDI-MSI results, we conducted AP-MALDI-MSI at a 5 × 5 μm^2^ pixel resolution on the PFA-fixed tissue. The intensity maps acquired by high-resolution AP-MALDI-MSI highlight the magnitude and heterogeneous spatial localization between Gb3 molecular lipoforms (Figure 3A). Whereas some of the Gb3 signals exhibit pronounced accumulation in selected regions of the tissue as highlighted by arrows in Figure 3A, Gb3 compounds differing solely in the fatty acyl composition have lower signal intensity in these respective regions and increased signal intensities in other areas of the tissue. This is best represented by the overlay image for selected examples shown in Figure 3B. Here, intensity maps of the differential spatial patterns of Gb3 (42:2) ([M + Na]^+^, *m*/*z* 1156.769, orange), Gb3 (38:1) ([M + Na]^+^, *m*/*z* 1102.722, blue), and the omnipresent lipid PC (34:1) ([M + Na]^+^, *m*/*z* 782.567, gray) are overlaid. Comparison to the BF and H&E images suggests that these regions of Gb3 accumulations are confined to one single or few neighboring cells in cardiac tissue (Figure 3B). Notably, the predominant molecular adducts differed between the 25 and 5 μm spatial resolution measurements, shifting from potassium (K^+^) adducts to sodium (Na^+^) adducts. This shift can be attributed to the PFA fixation procedure applied prior to the 5 μm measurements. Importantly, the total number of detected Gb3 species was not affected by the fixation process as documented in Figure S3.

**Figure 3:**
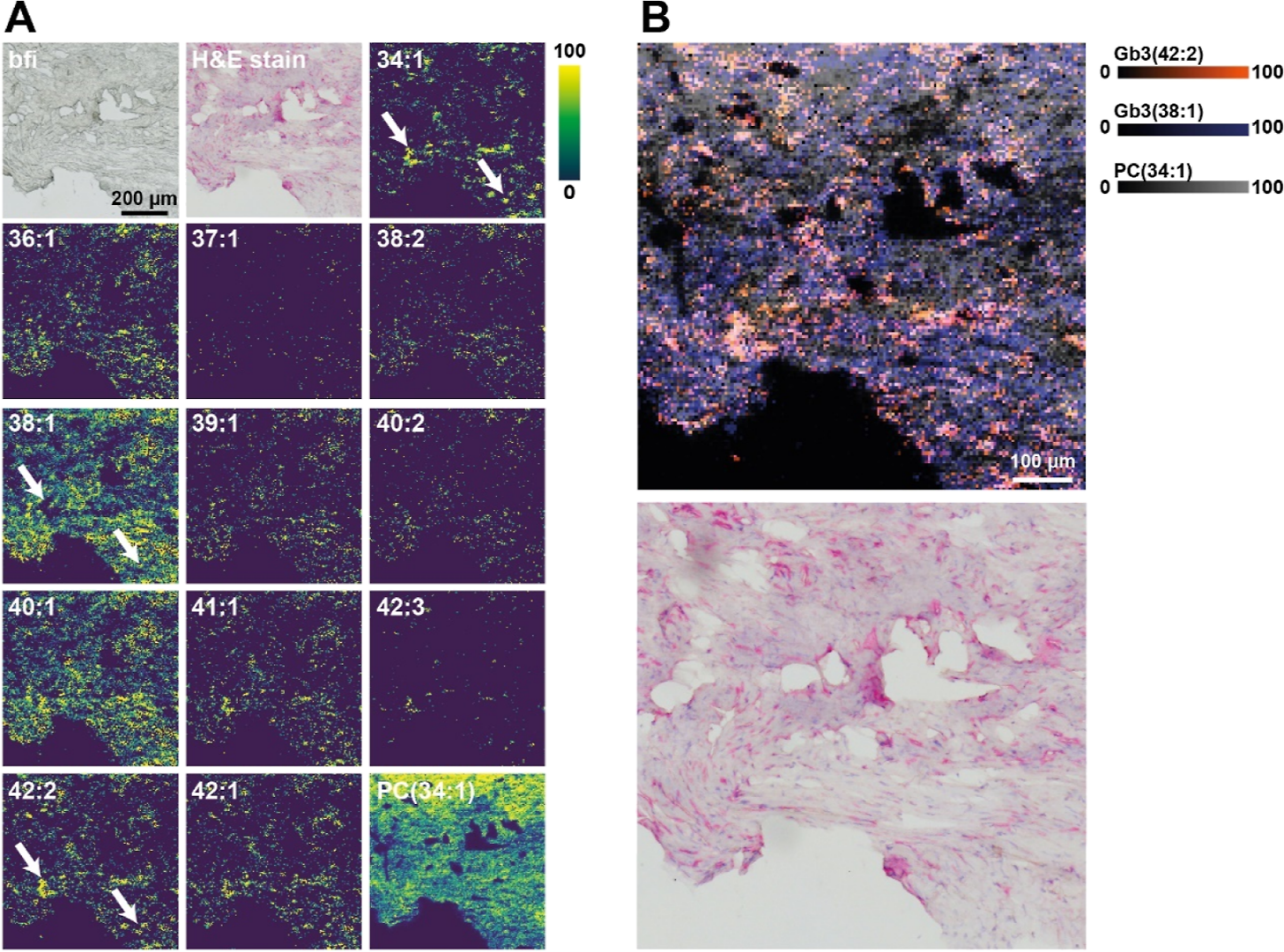
High-resolution AP-MALDI-MSI intensity maps of Gb3 species in a G3Stg/GLA^KO^ sample. (A) BF images of unstained and H&E stained tissues and the intensity distributions of all detected Gb3 species as [M + Na]^+^-adducts are shown. Data were recorded after formalin fixation and Raman microscopy acquisition from CaF_2_-glass discs with a 5 × 5 μm^2^ pixel size in spot acquisition mode. Annotation was performed based on the accurate mass of [M + Na]^+^-adducts and MS/MS analysis. (B) Overlay of Gb3 (42:2) ([M + Na]^+^, *m*/*z* 1156.7691, orange), Gb3 (38:1) ([M + Na]^+^, *m*/*z* 1102.7223, blue), and PC (34:1) ([M + Na]^+^, *m*/*z* 782.5673, gray) and the corresponding bright-field image of the H&E stained tissue. Relative intensity values for ion image intensity scale bars are shown in %.

Our high-resolution MALDI-MSI data, visualizing the different intensity distributions based on Gb3 lipoforms, are in line with previous findings by Onoue et al. where they manually correlated the localization of Gb3 species with vacuolar degeneration in cardiomyocytes of human endocardial biopsy samples.^51^ The heterogeneous accumulation of Gb3 in the heart tissue of FD models is known from Gb3 staining and has been described for different cardiac cell types such as cardiomyocytes, endothelial cells and smooth muscle cells of vessels, conduction tissue or fibroblasts.^10,51–56^ However, the distinct molecular composition and the heterogeneity between these lipid compounds are not available from staining experiments. Despite the existence of several studies that employed MALDI-MSI for the characterization of Gb3 accumulation in FD in a variety of tissues including kidney, heart, and skin,^51,57–60^ until now no data with comparable degree of molecular detail and spatial resolution for the determination of FD-associated Gb3 accumulation in heart tissue are available. As the focus of the present study was the automated coregistration of two bioanalytical imaging modalities with low pixel size, a comprehensive biological evaluation of Gb3 signal distributions in conjunction with post-MALDI-MSI H&E staining was not performed. This was not feasible within the scope of the study, as 20 μm thick tissue sections were employed, which did not permit reliable histological analysis on the single cell level.

### Raman Imaging of Heart Tissue from Murine FD Models

For Raman microscopy, the images of mouse heart sections of the three genotypes were acquired and analyzed first applying HCA analyses (Figures 4 and S4A). The number of HCA clusters set to 10 allowed a separation of DNA/RNA accumulation, typically associated with nuclei or mitochondria, and the tissue matrix (Figure 4A). The “tissue” clusters were characterized by Raman intensity peaks at 1003 cm^–1^ representing phenylalanine, at 1255 cm^–1^ representing amide III band, and at 1445 cm^–1^ representing CH_2_/CH_3_ stretching.^61,62^ The DNA/RNA (“nucleus”) clusters consisted of Raman intensity peaks at 785 cm^–1^ and 1095 cm^–1^ characteristic for DNA backbone and nucleic acids.^61^ For verification of the association of the assigned cluster with nuclei, additional H&E staining was performed (Figure S4B). The localization of these clusters is presented on the color maps (Figure 4C, left column; “nucleus” in blue; “tissue” in green). The distribution and number of pixels of these clusters were comparable in all genotypes.

**Figure 4:**
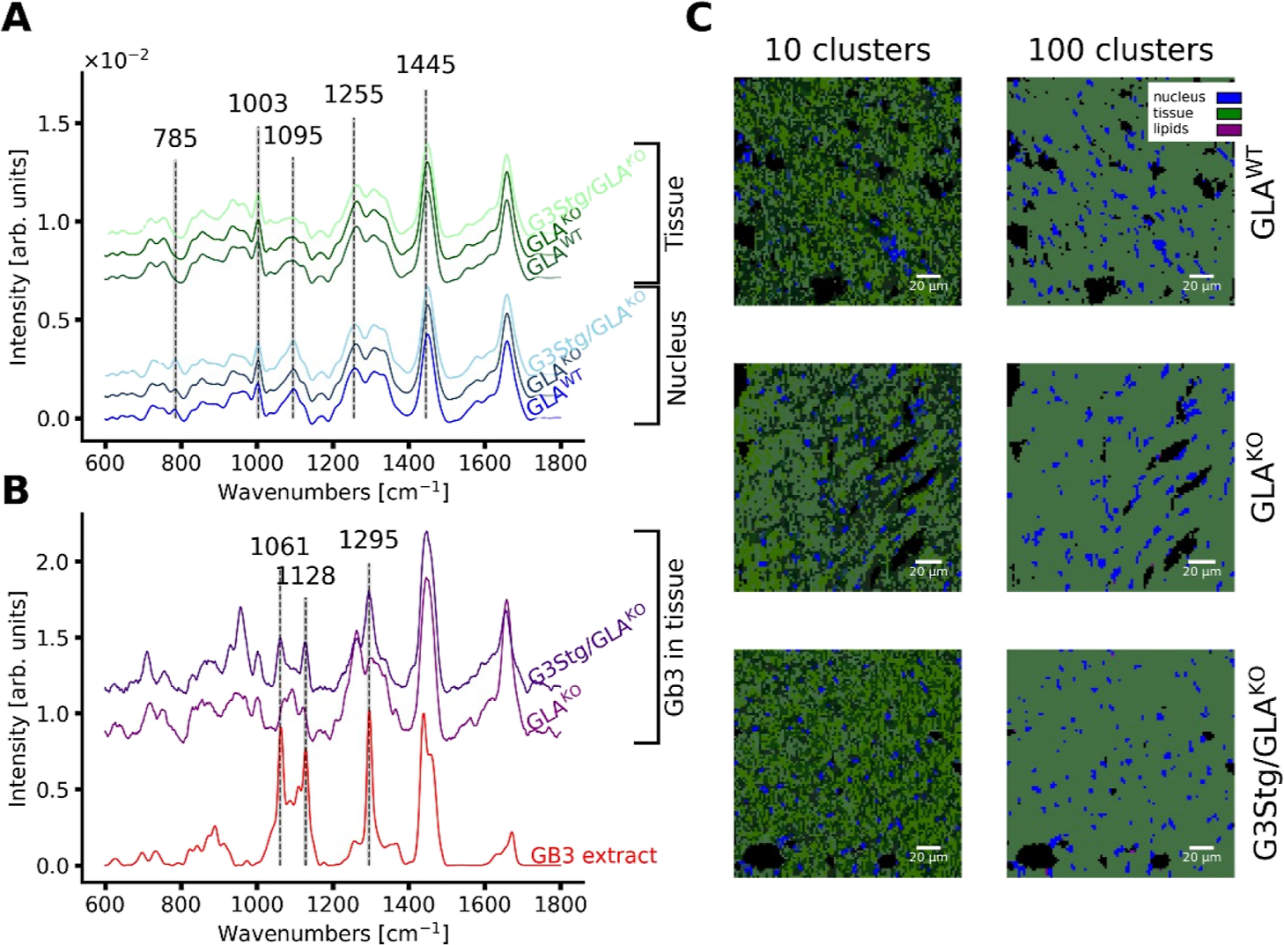
Multicluster hierarchical clustering analysis (HCA) of the Raman imaging of tissue section. (A) Mean spectra of the cluster analyses with 10 clusters. For each genotype, the mean spectrum of the nucleus and the mean spectrum of the remaining tissue were computed. The main intensity peaks (dashed lines) of nucleus and biological tissue components are visualized. Spectra are shifted along the *y*-axis by 25% of each spectrum’s maximum intensity for visual clarity. (B) Mean spectra of clusters derived in HCA analyses with 100 clusters and associated with Gb3 compounds detected in both GLA^KO^ and G3Stg/GLA^KO^ samples (based on reference spectrum of commercial Gb3 mixture). (C) Cluster images of the corresponding HCA are shown for 10 and 100 clusters per genotype, respectively. Nuclei-dense regions are depicted in blue; tissue is shaded in green, Gb3 is shaded in purple. Regions shown in black were discarded by the background segmentation of the preprocessing based on out-of-focus spectra or structural gaps within the tissue and were not included in the clustering process.

Aiming to distinguish additional chemical components within the tissue, the number of clusters was increased to 100. Figure 4B shows two additional clusters characterized by spectra with intense Raman peaks at 1061 cm^–1^, 1125 cm^–1^, and 1297 cm^–1^. Those peaks correlate with the characteristic intensity peaks of a commercial Gb3 extract, of which a reference spectrum is shown in Figure 4B. The Gb3 clusters were localized only in a few pixels of tissue sections (Figure 4C, right column, in purple). Even though Raman peaks of the purified Gb3 extract and of certain pixels in the tissue sections correlate, the identification of Gb3 by Raman imaging in tissue is challenging.

In order to characterize further molecular species using Raman imaging, we applied MCR-ALS to extract chemical components of Raman spectra and their corresponding relative abundances. The overview of all computed components found in the tissue is shown in the Supporting Information (Figures S5 and S6). After MCR-ALS analysis, we selected four main components either based on their contribution to the overall signal or on biological relevance.

The distribution of the selected four components in the tissue of the three genotypes is shown in Figure 5A with corresponding spectra depicted in Figure 5B. A detailed list of the band association for the components in Figure 5 can be found in Table S2. The “nucleus” component revealed homogeneously distributed DNA spots in tissues with similar abundance throughout the three genotypes. The “tissue” component showed the morphological structure of the tissue, mainly based on the lipid/protein ratio. Additionally, the analysis identified a “collagen” component (intensity peaks at 858 cm^–1^, 947 cm^–1^, and 1240 cm^–1^),^63,64^ highlighting the ability of Raman imaging to detect biologically relevant components that are not routinely captured with MALDI-MSI. Finally, the “lipid” component (intensity peaks at 1064 cm^–1^ and 1127 cm^–1^ (νC–C region), 1265 cm^–1^ (=CH), 1295 cm^–1^ (CH_2_) and 1439 cm^–1^ (CH_2_/CH_3_))^19,65,66^ revealed localized accumulations throughout the tissue with the highest abundance found in G3Stg/GLA^KO^. As the Gb3 clusters were not routinely separated from other components due to the spectral similarity with other lipids, a lipid-associated cluster was defined as a “lipids” group, shown in Figure 5A. Importantly, lipid abundance measured by Raman microscopy was significantly higher in G3Stg/GLA^KO^ cardiac tissue compared to both GLA^KO^ and GLA^WT^ mice, whereas just an average increase in the lipid content but no significant difference was observed between the GLA^KO^ and GLA^WT^ groups.

**Figure 5:**
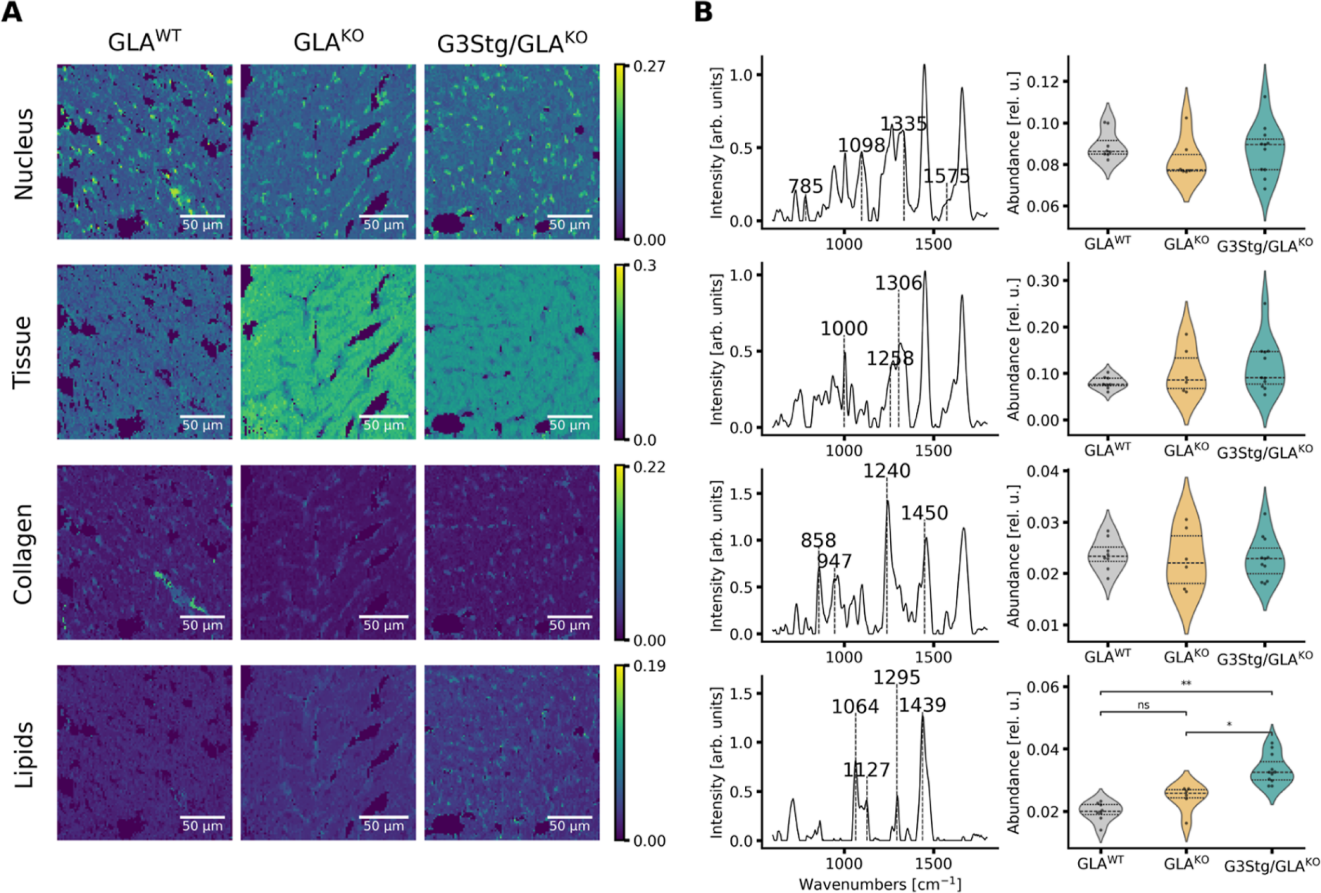
Component identification between different disease stages using HCA + MCR-ALS. (A) The abundance maps of DNA-rich component (“nucleus”), a protein-rich component, (“tissue”), collagen, and lipids for a typical tissue example of GLA^WT^, GLA^KO^, and G3Stg/GLA^KO^. Images were acquired with a 2 μm pixel resolution. Each intensity map was normalized to maximum intensity of this component over all measurements. (B) The component spectra and the corresponding distribution of the mean abundance. The distribution plots show the mean summed intensity for each component. Statistical significance was tested on the mean intensity (derived from the mean abundance of the measurements) and visualized in case of significance. **, *p* < 0.01; *; *p* < 0.05; ns = not significant.

Raman imaging enabled a chemical component map of the cardiac tissue with various components: nucleic acids, lipids, proteins, and collagen, and their relative abundance within the tissue. Whereas AP-MALDI-MSI allowed the detection of the disease-specific lipid Gb3 and enabled the statistical differentiation among all three genotypes, Raman imaging offered the analysis and detection of a broader range of chemical components and revealed trends in overall lipid intensities that were consistent with MSI-based observations for Gb3 lipids.

### Automated Coregistration of AP-MALDI-MS and Raman Imaging of Mouse Heart Tissue

Next, we aimed to integrate the complementary techniques, Raman imaging, and AP-MALDI-MSI at the micrometer scale to maximize their combined analytical capabilities. The respective results are shown in Figure 6. For this integration, a software application for the automated coregistration of Raman imaging and AP-MALDI-MSI data sets was developed. First, the Raman and MALDI-MSI results were overlaid with the corresponding BF images. For Raman imaging, BF was acquired at the same microscope, enabling accurate identification of areas measured by Raman within the BF overview image. For MALDI-MSI, BF images were acquired by using an external microscope before and after each measurement. To map MALDI-MSI frames onto the BF overview image acquired by the external microscope, we identified regions where the MALDI laser had irradiated the tissue, as indicated by color changes in the BF image. Thus, the areas on the tissue that displayed changes in BF coloration were matched with the corresponding MALDI data. Subsequently, the BF-mapped Raman imaging and MALDI-MSI data sets were overlaid by spatially aligning the Raman- and MALDI-associated BF images. Examples of the individual steps involved in this process are shown in Figure S7.

**Figure 6:**
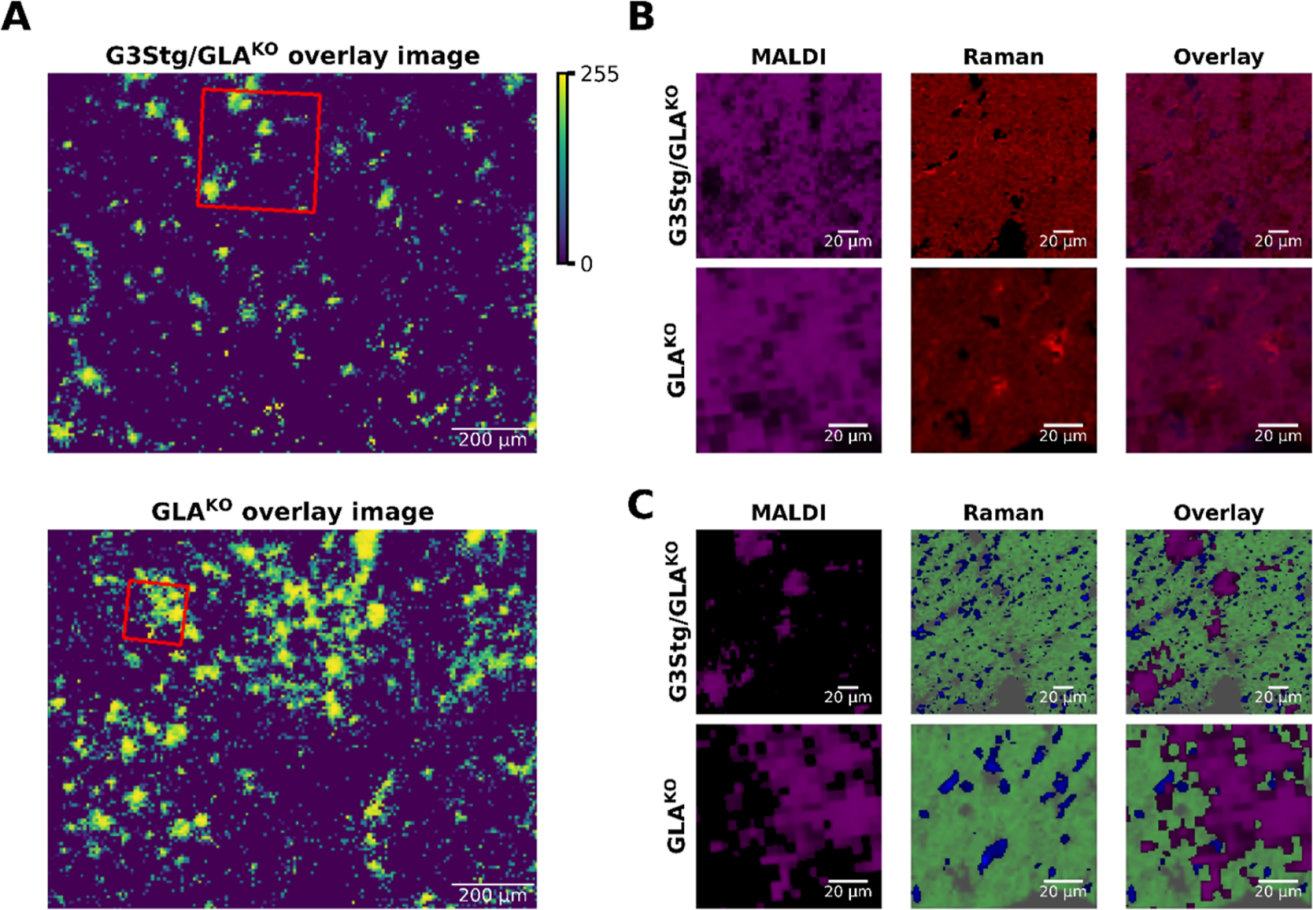
Automated overlay of AP-MALDI-MSI and Raman images. (A) RS measurements were located at their corresponding positions in the AP-MALDI-MSI overview tissue measurement of Gb3 (34:1) + Na]^+^ (*m*/*z* 1046.6591) for both G3Stg/GLA^KO^ and GLA^KO^ genotypes. (B) [PC 34:1 + Na]^+^ (*m*/*z* 782.5673) from AP-MALDI-MSI results, Raman color map of the lipid component, and the overlay of both modalities. (C) [Gb3(34:1) + Na]^+^ (*m*/*z* 1046.6591) accumulations (visualized in purple) from AP-MALDI-MSI overlaid with “tissue” component in green and “nucleus” component in blue derived by MCR-ALS from Raman measurements. Scale: 20 μm.

In order to evaluate the alignment of Raman and MALDI-MSI measurements, we focused on the three necessary tasks in the procedure: (1) the localization of Raman imaging results in the Raman-associated BF image, (2) the localization of AP-MALDI-MSI measurements in the MALDI-associated BF image, and (3) the coregistration of Raman- and MALDI-associated BF images. Tasks (1) and (2) were evaluated by calculating the intersection over Union (IoU) between the computed rectangle coordinates and manually set reference boxes for both tasks.^67^ This resulted in IoU scores of 0.985 and 0.991 for AP-MALDI-MSI and Raman imaging, respectively. These results indicate that automated localization of Raman imaging and MALDI-MSI within corresponding BF images achieves comparable registration accuracy compared to manual area selection. For step (3), we evaluated the automatic coregistration performance by measuring distances between reference points in both BF images. This approach achieved an automated coregistration accuracy of (5.1 ± 1.6) μm for aligning Raman imaging and AP-MALDI-MSI data, which is comparable to reported metrics for MALDI-MSI localization methods in histopathological BF images^68^ but here enables the overlay of two complementary bioanalytical imaging techniques. Representative examples of the evaluation are visualized in Figure S8. Our results indicate that coregistration of two high-resolution imaging modalities on pixel level can be achieved with our bioanalytical pipeline; however, cellular resolution and below was not within the scope of the present work.

Figure 6 illustrates coregistration results of Raman imaging and AP-MALDI-MSI for both G3Stg/GLA^KO^ and GLA^KO^. In Figure 6A, Raman measurements (small red squares) are overlaid with the corresponding sodiated Gb3 (34:1) overview images from AP-MALDI-MSI.

To validate the coregistration performance of the automated overlay between Raman and AP-MALDI-MSI, the distribution of the ubiquitous sodiated lipid PC (34:1) detected by AP-MALDI-MSI was compared to the lipid component distribution obtained from Raman imaging (Figures 6B and S9). Finally, we integrated the Gb3 distributions from AP-MALDI-MSI (visualized in purple) with additional biological components derived from Raman imaging, namely, the cardiac tissue (green) and nucleic acids (blue) (Figure 6C). This multimodal data integration demonstrates the complementary analytical capabilities of both techniques. Raman imaging characterizes the morphological and biochemical structure of cardiac tissue by visualizing nuclei and tissue architecture including general lipid distribution, while AP-MALDI-MSI specifically detects FD-related Gb3 accumulations enabling molecular differentiation.

## Conclusion and Outlook

We have developed a bioanalytical pipeline for the integration of AP-MALDI-MSI and Raman spectroscopy at the low micrometer scale. While several seminal studies have combined Raman and MSI technologies,^27–30,69,70^ the results presented here represent the first integration of these two modalities that achieves coregistration of MSI data at a 5 μm pixel size with Raman microscopy at a 2 μm pixel size in preclinical mouse models.

We demonstrate that both methods independently capture significant changes in Gb3 and lipid accumulations, and that the combination of 5 μm—AP-MALDI-MSI with Raman spectroscopy reveals the heterogeneous landscape of Gb3 accumulations. In the GLA^KO^ and G3Stg/GLA^KO^ mouse FD models employed here, Gb3 accumulation within lysosomes and following lysosomal rupture in cardiac fibroblasts has been previously reported using fluorescence microscopy.^54,56^ The multimodal Raman/AP-MALDI-MSI workflow developed in this study now provides increased molecular resolution for Gb3 accumulation in cardiac tissue. This approach revealed that Gb3 aggregation is not only heterogeneously distributed across the tissue but also exhibits variable lipidomic composition. This additional layer of heterogeneity among different Gb3 isoforms is not accessible by fluorescence microscopy methods. The automatic coregistration of AP-MALDI-MSI, Raman imaging, and BF microscopy data with about a 5 μm accuracy enabled contextualization of Gb3 accumulation within the tissue. Consequently, the presented AP-MALDI-MSI method has the potential to significantly contribute to a better characterization of the heterogeneous organization of Gb3 in tissues of the FD.

These developments will enable studies with larger cohorts that investigate the functional consequences of the molecular and spatial heterogeneity of Gb3 aggregation within cardiac tissue and, thus, rationalize the pathomechanisms underpinning these heterogeneities. Whereas AP-MALDI-MSI revealed localized heterogeneous regulation of Gb3 in heart tissue so far undetected via microscopic methods, Raman imaging provided tissue context but did not reveal new insights into the tissue architecture or molecular regulation in this setting. Therefore, future applications of this workflow will be expanded to human biopsy material to correlate different Gb3 lipoforms with patient phenotypes and evaluate whether therapeutic outcomes are associated with altered lipidomic profiles of Gb3 in the cardiac tissue. Beyond FD, this pipeline has other potential applications in biomedical research where also Raman imaging can reveal biomolecular aggregation events, such as, for example, the analysis of the microenvironment in myocarditis or diet-dependent accumulation of lipids in cardiac tissue.^71,72^

## Supplementary Material



## Notes

H&E staining protocol, LC–MS/MS method description, Raman preprocessing method, LC–MS/MS results, AP-MALDI-MSI results for Gb3 compound for different lipoforms, mean spectra and mean abundance of Raman results by MCR-ALS analysis for all genotypes, and representative coregistration results (PDF)
