# Supplementary material for: Automatic Coregistration of High-Resolution MALDI-MSI and Raman Imaging Applied to Cardiac Tissue of Fabry Disease Mouse Models
Source: Anal Chem. 2026 Jun 4;98(28):20725–37. doi: 10.1021/acs.analchem.5c07622 (PMC13393138; doi:10.1021/acs.analchem.5c07622)
Supplement: Supplementary file 1 [file ac5c07622_si_001.pdf]

# Supporting Information

## Automatic co-registration of high resolution MALDI-MSI and Raman imaging applied to cardiac tissue of Fabry disease mouse models

Johann Dierks[1,¥], Eike Ulrich Brockmann[1,¥], Anahi-Paula Arias-Loza[2], Thomas Bocklitz[3,4,5], Peter Nordbeck[6], Kristina Lorenz[1,7,*], Elena Tolstik[1,*], Sven Heiles[1,8,*]

1 Leibniz-Institut für Analytische Wissenschaften – ISAS – e.V., Bunsen-Kirchhoff Straße 11, 44139 Dortmund, Germany

2 Comprehensive Heart Failure Center, Department of Nuclear Medicine, University Hospital Würzburg, Am Schwarzenberg 15, 97078 Würzburg, Germany

3 Leibniz Institute of Photonic Technology e.V., Albert-Einstein-Straße 9, 07745 Jena, Germany

4 Institute of Physical Chemistry (IPC), Friedrich Schiller University Jena, Helmholtzweg 4, 07743 Jena, Germany

5 Abbe Center of Photonics (ACP), Albert-Einstein-Straße 6, 07745 Jena Germany

6 Department of Internal Medicine I, University Hospital Würzburg, Building A3/A4, Oberdürrbacher Str. 6, 97080 Würzburg, Germany

7 Institute of Pharmacology and Toxicology, Julius-Maximilians-University Würzburg, Versbacher Str. 9, 97078 Würzburg, Germany

8 Lipidomics, Faculty of Chemistry, University of Duisburg-Essen, Universitätsstr. 2, 45141 Essen, Germany

¥ Shared first authors

*Shared corresponding authors (Contact details: Kristina Lorenz, Kristina.Lorenz@isas.de; Elena Tolstik, Elena.Tolstik@isas.de; Sven Heiles, Sven.Heiles@isas.de)

## Table of Contents

Materials and Methods.......................................................................................................S3

Post-acquisition H&E staining .........................................................................................S3

LC-MS/MS of cardiac tissue extracts for lipid identification...................................................S3

Pre-processing of Raman results .......................................................................................S4

Results ................................................................................................................................S5

**Figure S1:** Heatmap of normalized mean ROI intensities: ...................................................S5

**Figure S2:** LC-MS/MS fragment spectra of all detected Gb3 species..................................S6

**Table S1**: List of detected Gb3Cer species as identified by accurate mass in AP-MALDI MSI and LC-MS/MS...........................................................................................................S7

**Figure S3**: Comparison of AP-MALDI-MS mass spectra for the Gb3 mass range.................S8

**Figure S4:** The dendrograms for the HCA clustering and H&E comparison. .........................S9

**Table S2:** Collection of Raman bands and their biochemical assignment detected in the HCA+MCR-ALS analysis. ..............................................................................................S10

**Figure S5:** Mean spectra for all computed 35 components found in the tissues and identified using HCA+MCR-ALS. .........................................................................................S11

**Figure S6:** Distribution of the mean abundance for all computed components found in the tissues and identified using MCR-ALS. ...............................................................S12

**Figure S7:** Example of the superimposition between MALDI, Raman and BF imaging. ......S13

**Figure S8:** Representative examples of the evaluation of the co-registration algorithm. .....S14

**Figure S9:** Overlay of lipid maps measured with AP-MALDI MS and Raman with BF images. ..........................................................................................................................S15

Literature ............................................................................................................................S16

# Materials and Methods

## Post-acquisition H&E staining

Hematoxylin and eosin (H&E) staining of tissue sections following MALDI-MSI acquisition was performed according to a standardized protocol. Briefly, matrix-coated sections were washed in isopropanol for 2 minutes. Deparaffinization was then carried out using ROTI®Histol (6640.1, Carl Roth GmbH + Co. KG, Karlsruhe, Germany) in a three-step procedure, with each step lasting 4 min. Rehydration was performed sequentially using four washes of absolute ethanol and one wash of 70% ethanol, each for 2 min. Prior to staining, samples were rinsed in water four times, each rinse lasting 30 s.

Nuclear staining was conducted using Mayer's hematoxylin (MHS32-1L, Sigma-Aldrich, Merck KGaA, Darmstadt, Germany) for 10 min, followed by a bluing step in running tap water for an additional 10 min. Counterstaining was performed with eosin G (yellowish) (1.15935.0100, Merck KGaA, Darmstadt, Germany; 10 g/L in Milli-Q water) for 50 s. Excess stain was removed by sequential rinsing in five water cuvettes.

Dehydration was achieved using 70% ethanol for 1 min, followed by four successive washes in absolute ethanol, each lasting 2 min. Tissue clearing was performed with two changes of ROTI®Histol, 3 min each. Finally, stained sections were mounted using Eukitt® quick-hardening mounting medium (03989, Merck KGaA, Darmstadt, Germany) and allowed to dry overnight in a dark environment.

## LC-MS/MS of cardiac tissue extracts for lipid identification

Lipid extracts were prepared from five to seven 20 µm-thick mouse heart sections per experimental group using a combined approach based on the Matyash[1] and SIMPLEX[2] protocols. Samples were handled on ice whenever possible. All tissue sections from a given group were pooled into a single Eppendorf tube. Homogenization was carried out using the Bioruptor® Plus sonication device (Diagenode SA, Seraign (Ougrée), Belgium) by adding 200 µL of ice cold 0.1% ammonium acetate in Mili-Q water, 1 µL of SPLASH® Lipidomix®, 1 µL of Gb3 C17:0 (100 µg/mL), and six to eight protein extraction beads (C20000021, Diagenode SA, Seraign (Ougrée), Belgium) to the tissue sample. The samples were sonicated at 4 °C using an alternating cycle of 30 s sonication and 30 s cooling, for a total duration of 10 min.

Lipid extraction was carried out by adding 120 µL of ice-cold methanol (MeOH) followed by 540 µL of ice-cold methyl tert-butyl ether (MTBE) to the homogenized samples. The mixture was incubated for 1 h at 4 °C with continuous agitation at 1200 rpm. Phase separation was induced by the addition of 200 µL of 0.1% ammonium acetate in water. Samples were then centrifuged at 10,000 rpm for 10 min at 4 °C. The upper organic phase was carefully transferred to a clean Eppendorf tube. The remaining aqueous phase was subjected to a second extraction using the same procedure. Both organic phases were combined and the solvent was evaporated to dryness using a vacuum centrifuge. The dried lipid extracts were reconstituted in 50 µL of methanol/isopropanol (1:1, v/v) and subsequently analyzed by liquid chromatography–tandem mass spectrometry (LC-MS/MS).

LC-MS/MS analysis was conducted using a Vanquish Flex UHPLC system (Thermo Fisher Scientific, Bremen, Germany) coupled to an Orbitrap Exploris 240 mass spectrometer (Thermo

Fisher Scientific, Bremen, Germany). Chromatographic separation was achieved on an Ascentis® Express C18 column (150 × 2.1 mm, 2.7 µm particle size, 90 Å pore size; Supelco, Germany) using a 36-minute stepped gradient from 10% to 100% mobile phase B.

Mobile phase A consisted of acetonitrile/water (1:1, v/v)) supplemented with 5 mM ammonium formate and 0.1% formic acid. Mobile phase B consisted of isopropanol/acetonitrile/water (85:10:5, v/v/v) with 5 mM ammonium formate and 0.1% formic acid. The gradient profile was as follows: 0–20 min, 10–86% B; 20–22 min, 86–100% B; 22–27 min, 100% B; 27–28 min, 100–10% B; 28–36 min, 10% B. The flow rate was set to 300 µL/min, and the column temperature was maintained at 50 °C. The injection volume was set to 5 µL.

Mass Spectrometric Parameters were as follows: The total method duration was 34 min. The H-ESI parameters were configured as follows: a static spray voltage of 3.1 kV, sheath gas flow rate of 40 (arbitrary units), auxiliary gas flow rate of 15, and sweep gas flow rate of 12. The ion transfer tube was maintained at a temperature of 320 °C, while the vaporizer temperature was set to 300 °C. The expected LC peak width was set to 30 seconds, and advanced peak determination was enabled. The default charge state was assigned as +1, and lock mass correction was applied using the EASY-ICTM system.

Full-scan mass resolution was set to 180,000, and for data-dependent MS/MS (ddMS2) scans, a resolution of 15,000 was employed, covering an m/z range of 150–1200. The RF lens level was adjusted to 70%. The automatic gain control (AGC) target was set to standard, and the maximum injection time was operated in automatic mode. Data acquisition was performed in positive ionization mode with profile detection, utilizing one microscan and no source-induced fragmentation. The intensity threshold for precursor selection was set to 1000.

Dynamic exclusion was enabled in custom mode, with exclusion applied after a single occurrence and maintained for 15 s. The mass tolerance for precursor selection was ±5 ppm for both high and low masses, and isotopic peaks were excluded. Data-dependent acquisition (DDA) was configured to collect ten MS/MS scans per cycle, employing an isolation window of m/z 1.5 and normalized higher-energy collisional dissociation (HCD) collision energies of 20%, 25%, and 30%.

## Pre-processing of Raman results

Data analysis of all measurements was performed using in-house written algorithms implemented in python 3.12 using numpy 1.26[3], scipy[4] and scikit-learn[5] for computation and matplotlib[6] for visualization. First, the data were pre-processed using an automatic cosmic spike correction[7], wavenumber calibration to a 1 $cm^{-1}$ grid, baseline correction using sensitive iterative peak (SNIP) clipping of the pybaseline package[8,9], Savitzky-Golay smoothing, background segmentation using k-means clustering, and vector normalization.

# Results

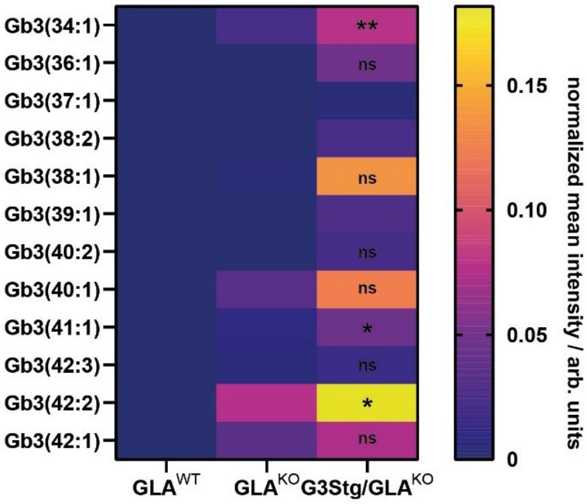


**Figure S1:** Heatmap of normalized mean ROI intensities:

The heatmap demonstrates the mean intensity differences within the groups for all Gb3 species as detected with AP-MALDI-MSI. The displayed intensities represent mean ROI intensities of the tissue area and were calculated following hot-spot removal by histogram equivalation and RMS normalization. $GLA^{WT}$ n=5, $GLA^{KO}$ n=4, G3Stg/$GLA^{KO}$ n=3 biological replicates. ** $p < 0.01$; * $p < 0.05$; ns = not significant. Significances were calculated as described in the methods section.

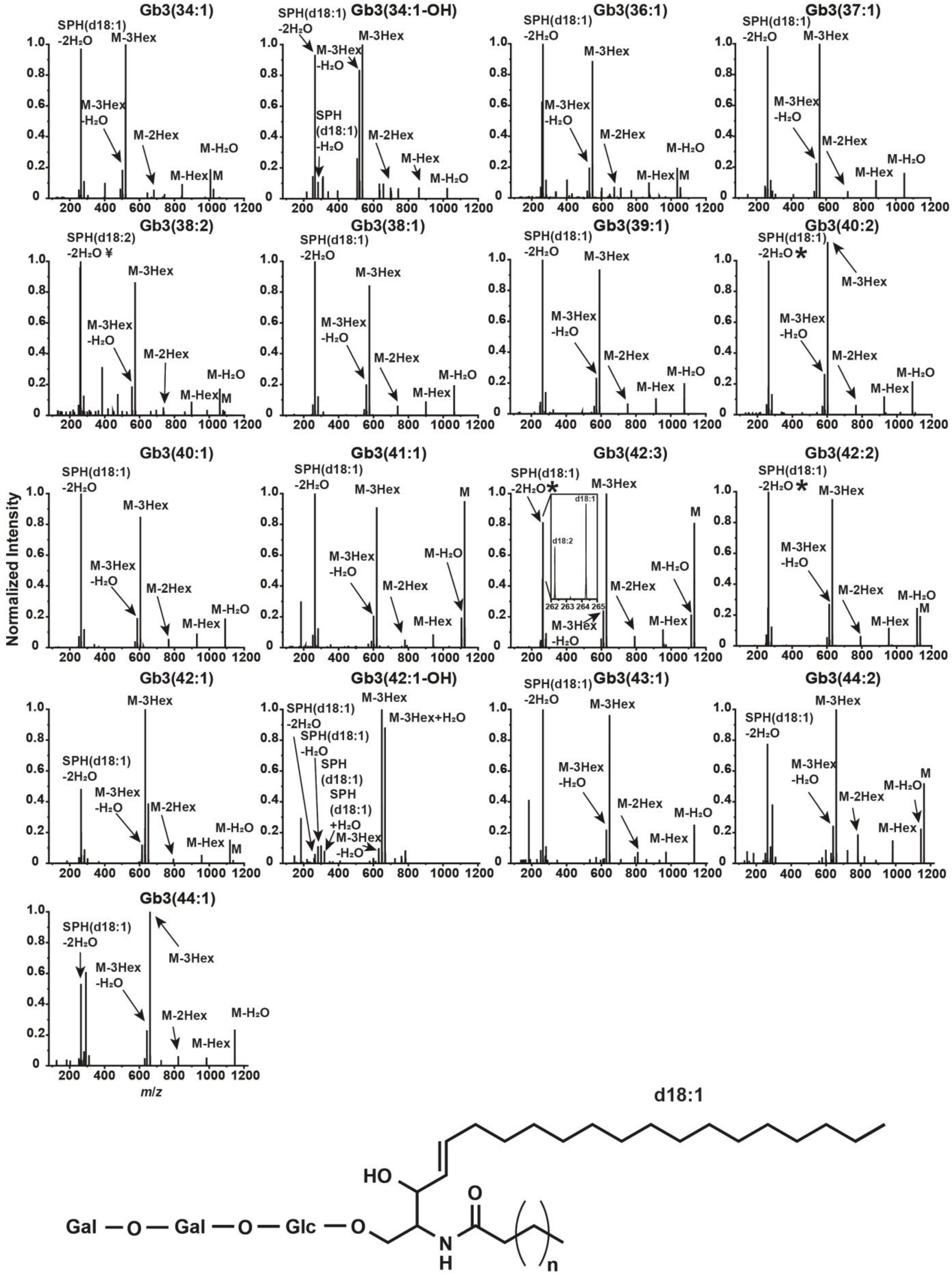


**Figure S2:** LC-MS/MS fragment spectra of all detected Gb3 species.

HCD of protonated Gb3 species resulted in distinct fragment spectra with a sequential loss of the three hexoses (Hex) and the additional cleavage of the fatty acyl chain and double water loss, resulting in the sphingosine moiety at m/z 264.26. * For Gb3(40:2), Gb3(42:2) and Gb3(42:3) the sphingadiene moiety (*m/z* 262.26) could be detected in addition to m/z 264.26. For Gb3(38:2), only the sphingadiene moiety could be detected.

**Table S1**: List of detected Gb3Cer species as identified by accurate mass in AP-MALDI MSI and LC-MS/MS.

| Gb3-Species | Sphingosine | Fatty acid | Exact mass $[M+H]^+$ | Δ (mDa) | ppm | LC-MS/MS | AP-MALDI-MSI $[M+Na]^+$, $[M+K]^+$ |
|---|---|---|---|---|---|---|---|
| **Gb3(d18:1/C16:0)** | d18:1 | C16:0 | 1024.6778 | 0.58 | 0.6 | x | x |
| **Gb3(d18:1/C16:0+OH)** | d18:1 | C16:0-OH | 1040.6727 | 0.58 | 0.6 | x | n/a |
| **Gb3(d18:1/C18:0)** | d18:1 | C18:0 | 1052.7091 | 0.18 | 0.2 | x | x |
| **Gb3(d18:1/C19:0)** | d18:1 | C19:0 | 1066.7248 | 0.32 | 0.3 | x | x |
| **Gb3(d18:2/C20:0)** | d18:2 | C20:0 | 1078.7248 | 0.58 | 0.5 | x | x |
| **Gb3(d18:1/C20:0)** | d18:1 | C20:0 | 1080.7404 | 1.02 | 0.9 | x | x |
| **Gb3(d18:1/C21:0)** | d18:1 | C21:0 | 1094.7561 | 1.02 | 0.9 | x | x |
| **Gb3(d18:2/C22:0)** | d18:2 | C22:0 | 1106.7561 | 0.22 | 0.2 | x | n/a |
| **Gb3(d18:1/C22:0)** | d18:1 | C22:0 | 1108.7717 | 0.52 | 0.5 | x | x |
| **Gb3(d18:1/C22:1)** | d18:1 | C22:1 | 1106.7561 | 1.22 | 1.1 | x | x |
| **Gb3(d18:1/C23:0)** | d18:1 | C23:0 | 1122.7874 | 0.32 | 0.3 | x | x |
| **Gb3(d18:2/C24:0)** | d18:2 | C24:0 | 1134.7874 | 0.82 | 0.7 | x | n/a |
| **Gb3(d18:1/C24:0)** | d18:1 | C24:0 | 1136.8030 | 0.12 | 0.1 | x | x |
| **Gb3(d18:1/C24:0+OH)** | d18:1 | C24:0-OH | 1152.7979 | 0.28 | 0.2 | x | - |
| **Gb3(d18:2/C24:1)** | d18:2 | C24:1 | 1132.7718 | 1.02 | 0.9 | x | n/a |
| **Gb3(d18:1/C24:1)** | d18:1 | C24:1 | 1134.7874 | 0.82 | 0.7 | x | x |
| **Gb3(d18:1/C24:2)** | d18:1 | C24:2 | 1132.7717 | 1.12 | 1 | x | x |
| **Gb3(d18:1/C25:0)** | d18:1 | C25:0 | 1150.8187 | 0.12 | 0.1 | x | - |
| **Gb3(d18:1/C26:0)** | d18:1 | C26:0 | 1164.8343 | 0.78 | 0.7 | x | - |
| **Gb3(d18:1/C26:1)** | d18:1 | C26:1 | 1162.8187 | 0.30 | 0.2 | x | - |

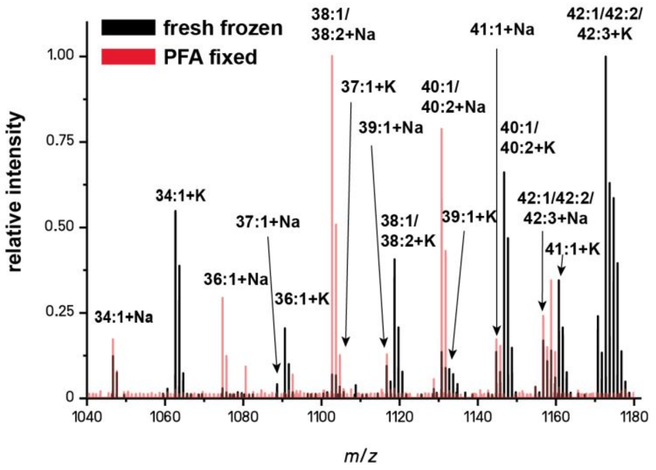


**Figure S3**: Comparison of AP-MALDI-MS mass spectra for the Gb3 mass range.

Red: Averaged spectrum of 25 scans in spot mode with 5 µm pixel size on PFA fixated GLAKO/G3Stg heart tissue. Black: Single pixel mass spectrum of a Gb3 hot-spot with 25 µm pixel size in full pixel mode on fresh-frozen GLAKO/G3Stg heart tissue. The labels represent the total number of carbons and double bonds of the sphingosine base and the fatty acyl chain plus the respective adduct.

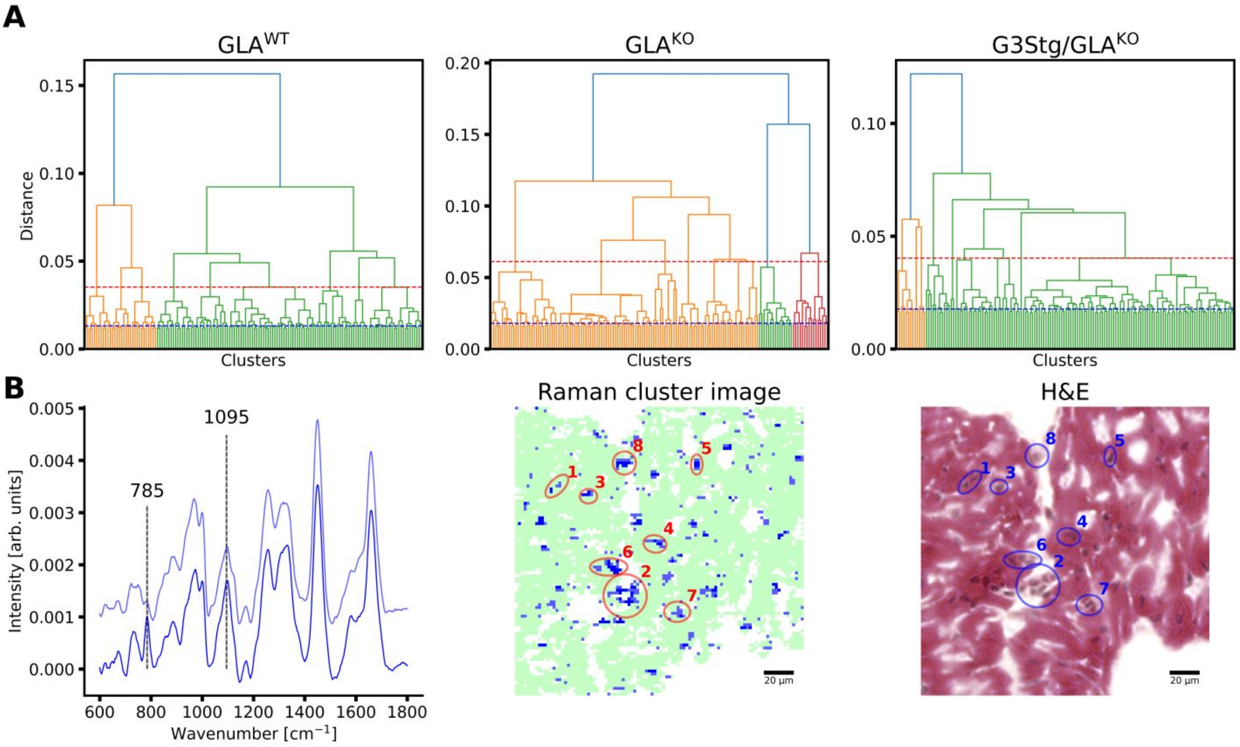


**Figure S4:** The dendrograms for the HCA clustering and H&E comparison.

**A)** The different dendrograms of each clustering shown in Figure 4. For visualization purpose, only the final 150 merging steps are shown. In the manuscript, cluster maps with n_clusters equals 10 and n_clusters equals 100 are shown. 10 clusters (red dashed line) were the minimal number to receive reliable identification of DNA/RNA-associated regions in all HCAs. Setting the cluster number to 100 (blue dashed line) was based on two criteria. First, it resulted in the distinction of lipid-associated clusters based on the resulting mean spectra in G3Stg/GLAKO and GLAKO scans. Secondly, setting the number of clusters relatively high, this ensured the creation of a dataset for the following HCA+MCR-ALS analysis, which was also capturing components with low occurrence. **B)** One example of an HCA clustering (10 clusters) compared to the corresponding H&E image. DNA/RNA-associated regions were identified in two clusters based on their respective mean spectra and band appearance at 785 $cm^{-1}$ and 1095 $cm^{-1}$ (left image). Similar regions in the cluster map (middle image, red circles) and corresponding H&E image (right image, blue circles) shows the overlay between DNA/RNA-associated regions and nuclei.

**Table S2:** Collection of Raman bands and their biochemical assignment detected in the HCA+MCR-ALS analysis.

Positions of the bands in the component spectra were determined using the find _peaks function of the scipy.signal python package. Assignment of the components in Figure 5 was done manually based on all detected peaks and their component assignment in the literature.

| Band position in $cm^{-1}$ (in the Manuscript) | Component assignment | Chemical assignment | References in Suppl. Material | Band position in $cm^{-1}$ (in References) |
|---|---|---|---|---|
| 785 | Nucleus | O-P-O stretching and ring breathing modes from nucleic acids | 10 | 785-788 |
| 858 | Collagen | Proline | 11 | 856 |
| 947 | Collagen | C-C-O | 11 | 943 |
| 1002 | Tissue | Phenylalanine, proteins | 10,12 | 1000-1004 |
| 1064 | Lipids | C-C stretch | 12 | 1061-1065 |
| 1098 | Nucleus | Symmetric $PO_2$ stretching | 10 | 1096-1097 |
| 1127 | Lipids | C-C | 12 | 1122-1130 |
| 1240 | Collagen | Amide III | 12 | 1220-1300 |
| 1258 | Tissue | Amide III | 12 | 1220-1300 |
| 1295 | Lipids | $CH_2$ deformation | 12 | 1296 |
| 1335 | Nucleus | Guanine | 10 | 1336 |
| 1439 | Lipids | C-H bending modes | 10 | 1420-1460 |
| 1450 | Tissue | $CH_2/CH_3$ deformations (protein, lipids) | 12 | 1445-1453 |
| 1575 | Nucleus | C=C ring breathing modes of adenine and guanine | 10 | 1572-1576 |

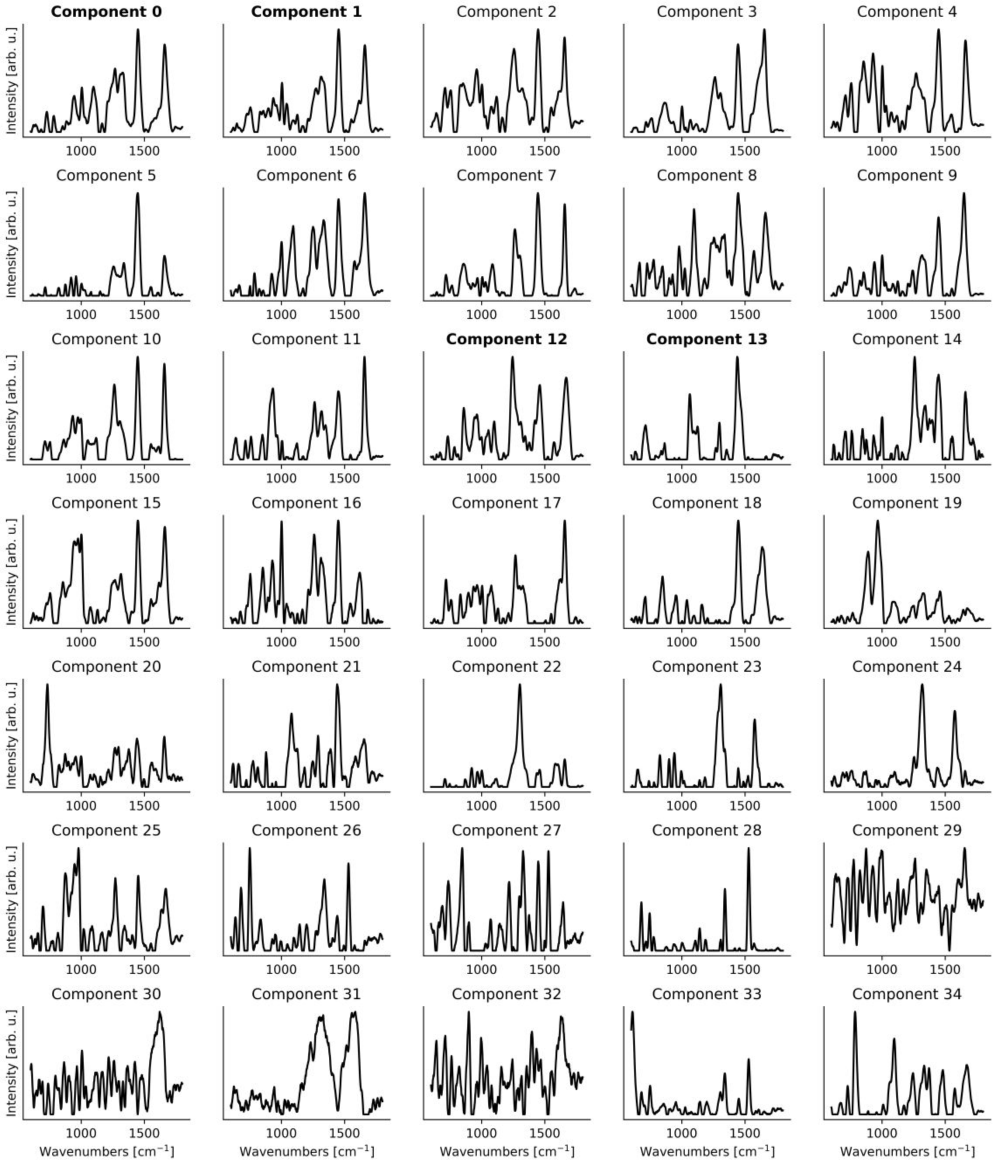


**Figure S5:** Mean spectra for all computed 35 components found in the tissues and identified using HCA+MCR-ALS.

The components are sorted by frequency of occurrence in the tissue. From all 35 components only four main components were selected for further study either based on their contribution to the overall signal or by biological relevance, see also **Figure S4**.

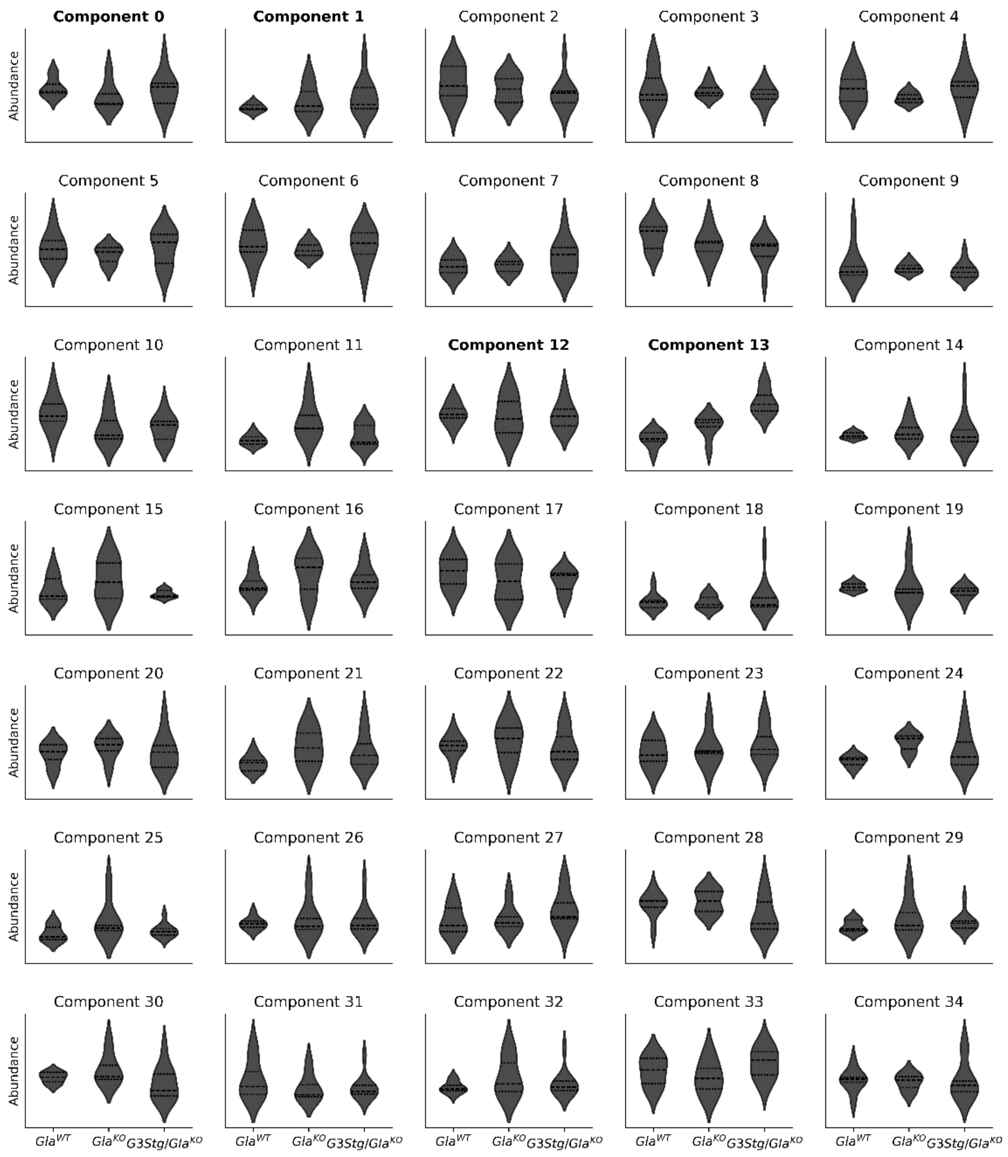


**Figure S6:** Distribution of the mean abundance for all computed components found in the tissues and identified using MCR-ALS.

The components are sorted by frequency of occurrence and each component is presented for three genotypes: $GLA^{WT}$, $GLA^{KO}$ and $G3Stg/GLA^{KO}$. From all 35 components only four main components were selected for further study either based on their contribution to the overall signal or by biological relevance.

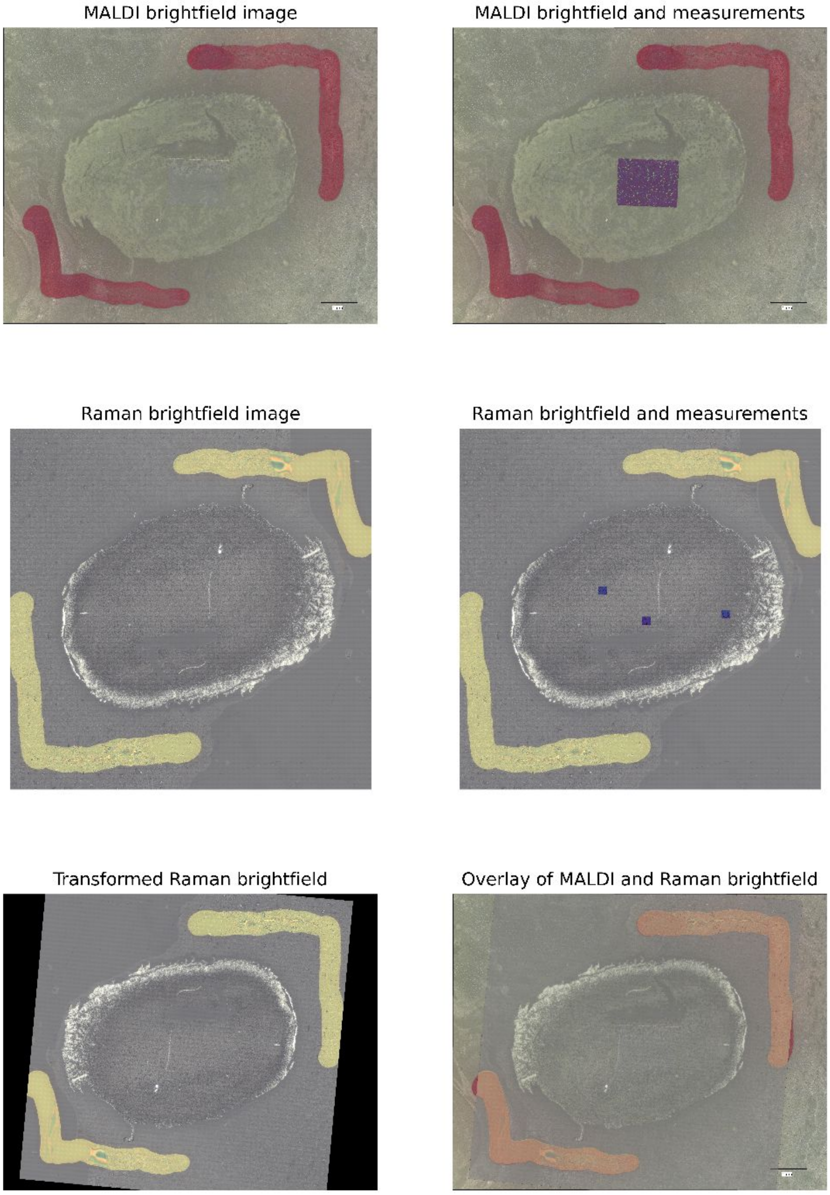


**Figure S7:** Example of the superimposition between MALDI, Raman, and BF imaging.

The two overview BF images are labelled “MALDI brightfield image” and “Raman brightfield image”. Localization of MALDI and Raman results in the BF images, as described for defined task (1) and (2), are shown on the right and are labelled “MALDI brightfield and measurement” and “Raman brightfield and measurement”, respectively. Coalignment of both BF images, task (3), and therefore coalignment of MALDI and Raman results, are shown in the lower row and are labelled “Transformed Raman brightfield” and “Overlay of MALDI and Raman brightfield”.

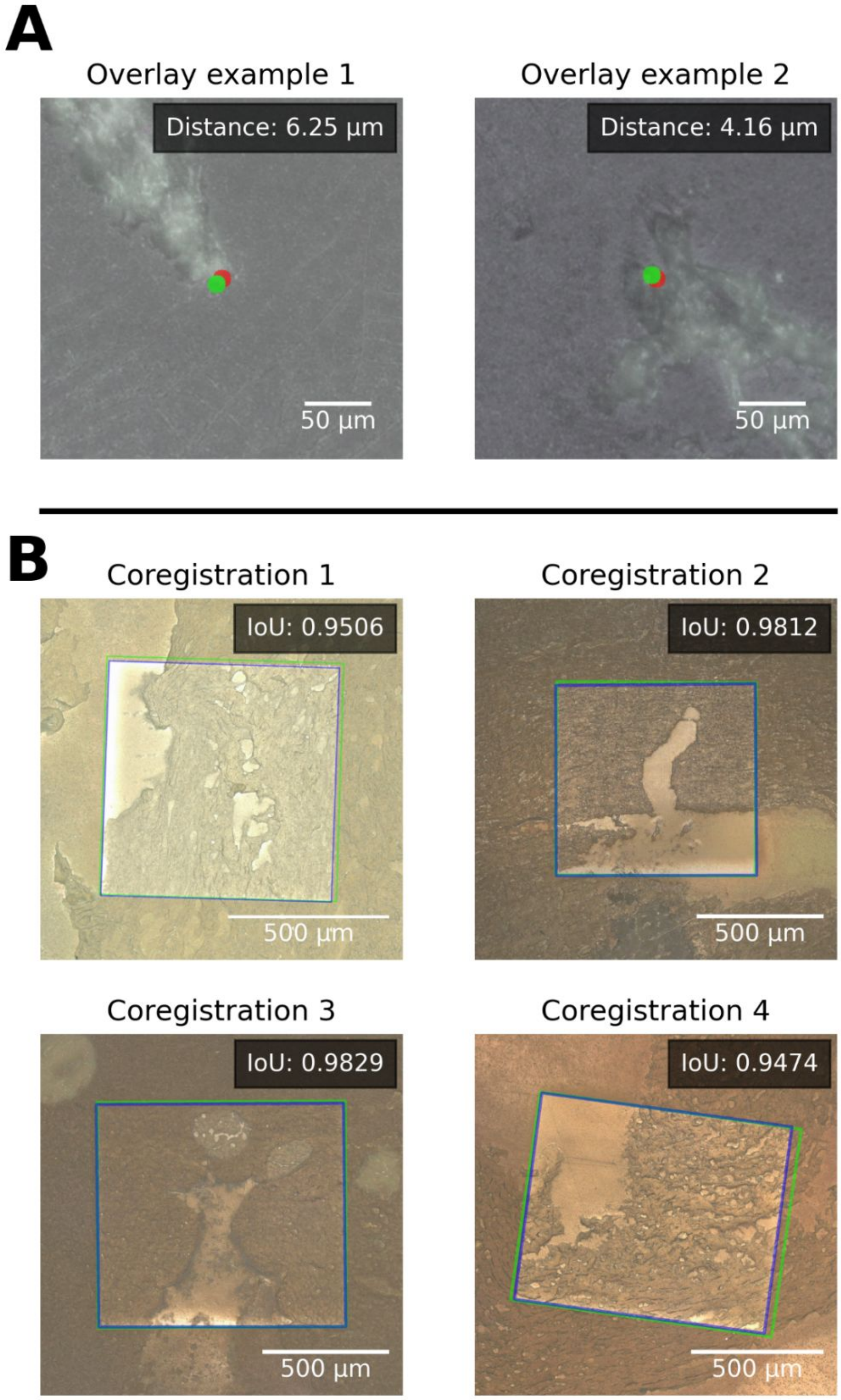


**Figure S8:** Representative examples of the evaluation of the co-registration algorithm.

**A)** Representative example of two co-registrations between the overview BF images. For both examples, a region of interest is shown, with the red dot representing the marker in the original MALDI BF image and the green dot representing approximately the same point marked in the original, untransformed Raman-associated BF overview. After transformation, distance between both dots were measured to estimate the transformation accuracy. **B)** Localization of AP-MALDI MS measurements (green rectangles) in the corresponding BF image based on intensity differences. The Intersection over Union (IoU) between the prediction and a ground truth (blue rectangle) was calculated. Thereby, the definition of the ground truth was done by approximating the polygon with different intensity left after AP-MALDI measurement in the BF image.

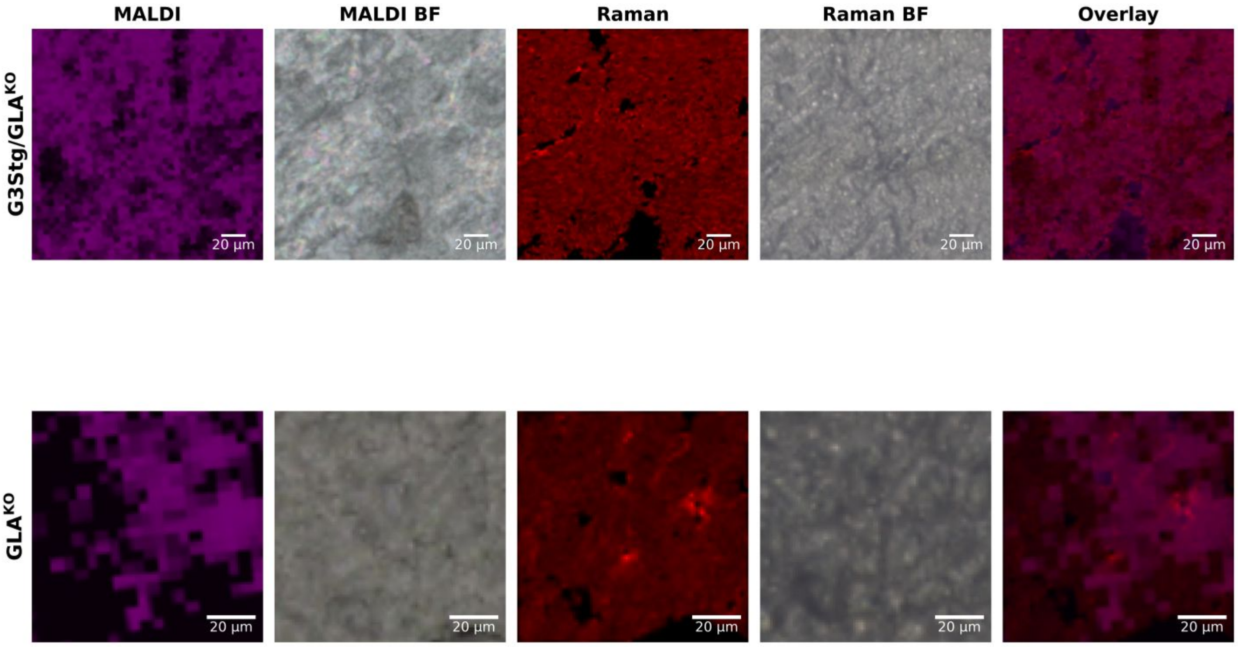


**Figure S9:** Overlay of lipid maps measured with AP-MALDI MS and Raman with BF images.

The general lipid species of AP-MALDI and its post-acquisition BF images were compared with the lipid component derived by HCA+MCR-ALS applied on Raman data and the BF image acquired at the Raman system. No visible similarities between the two BF modalities can be obtained as the AP-MALDI MS measurement alternates the tissue section and the two BF imaging modalities differs in their respective imaging setup.

# Literature


(1) Matyash, V.; Liebisch, G.; Kurzchalia, T. V.; Shevchenko, A.; Schwudke, D. Lipid Extraction by Methyl-Tert-Butyl Ether for High-Throughput Lipidomics. *J. Lipid Res.* **2008**, *49* (5), 1137–1146. https://doi.org/10.1194/jlr.D700041-JLR200.

(2) Coman, C.; Solari, F. A.; Hentschel, A.; Sickmann, A.; Zahedi, R. P.; Ahrends, R. Simultaneous Metabolite, Protein, Lipid Extraction (SIMPLEX): A Combinatorial Multimolecular Omics Approach for Systems Biology. *Mol. Cell. Proteomics MCP* **2016**, *15* (4), 1453–1466. https://doi.org/10.1074/mcp.M115.053702.

(3) Harris, C. R.; Millman, K. J.; Van Der Walt, S. J.; Gommers, R.; Virtanen, P.; Cournapeau, D.; Wieser, E.; Taylor, J.; Berg, S.; Smith, N. J.; Kern, R.; Picus, M.; Hoyer, S.; Van Kerkwijk, M. H.; Brett, M.; Haldane, A.; Del Río, J. F.; Wiebe, M.; Peterson, P.; Gérard-Marchant, P.; Sheppard, K.; Reddy, T.; Weckesser, W.; Abbasi, H.; Gohlke, C.; Oliphant, T. E. Array Programming with NumPy. *Nature* **2020**, *585* (7825), 357–362. https://doi.org/10.1038/s41586-020-2649-2.

(4) Virtanen, P.; Gommers, R.; Oliphant, T. E.; Haberland, M.; Reddy, T.; Cournapeau, D.; Burovski, E.; Peterson, P.; Weckesser, W.; Bright, J.; Van Der Walt, S. J.; Brett, M.; Wilson, J.; Millman, K. J.; Mayorov, N.; Nelson, A. R. J.; Jones, E.; Kern, R.; Larson, E.; Carey, C. J.; Polat, İ.; Feng, Y.; Moore, E. W.; VanderPlas, J.; Laxalde, D.; Perktold, J.; Cimrman, R.; Henriksen, I.; Quintero, E. A.; Harris, C. R.; Archibald, A. M.; Ribeiro, A. H.; Pedregosa, F.; Van Mulbregt, P.; SciPy 1.0 Contributors; Vijaykumar, A.; Bardelli, A. P.; Rothberg, A.; Hilboll, A.; Kloeckner, A.; Scopatz, A.; Lee, A.; Rokem, A.; Woods, C. N.; Fulton, C.; Masson, C.; Häggström, C.; Fitzgerald, C.; Nicholson, D. A.; Hagen, D. R.; Pasechnik, D. V.; Olivetti, E.; Martin, E.; Wieser, E.; Silva, F.; Lenders, F.; Wilhelm, F.; Young, G.; Price, G. A.; Ingold, G.-L.; Allen, G. E.; Lee, G. R.; Audren, H.; Probst, I.; Dietrich, J. P.; Silterra, J.; Webber, J. T.; Slavič, J.; Nothman, J.; Buchner, J.; Kulick, J.; Schönberger, J. L.; De Miranda Cardoso, J. V.; Reimer, J.; Harrington, J.; Rodríguez, J. L. C.; Nunez-Iglesias, J.; Kuczynski, J.; Tritz, K.; Thoma, M.; Newville, M.; Kümmerer, M.; Bolingbroke, M.; Tartre, M.; Pak, M.; Smith, N. J.; Nowaczyk, N.; Shebanov, N.; Pavlyk, O.; Brodtkorb, P. A.; Lee, P.; McGibbon, R. T.; Feldbauer, R.; Lewis, S.; Tygier, S.; Sievert, S.; Vigna, S.; Peterson, S.; More, S.; Pudlik, T.; Oshima, T.; Pingel, T. J.; Robitaille, T. P.; Spura, T.; Jones, T. R.; Cera, T.; Leslie, T.; Zito, T.; Krauss, T.; Upadhyay, U.; Halchenko, Y. O.; Vázquez-Baeza, Y. SciPy 1.0: Fundamental Algorithms for Scientific Computing in Python. *Nat. Methods* **2020**, *17* (3), 261–272. https://doi.org/10.1038/s41592-019-0686-2.

(5) Pedregosa, F.; Pedregosa, F.; Varoquaux, G.; Varoquaux, G.; Org, N.; Gramfort, A.; Gramfort, A.; Michel, V.; Michel, V.; Fr, L.; Thirion, B.; Thirion, B.; Grisel, O.; Grisel, O.; Blondel, M.; Prettenhofer, P.; Prettenhofer, P.; Weiss, R.; Dubourg, V.; Dubourg, V.; Vanderplas, J.; Passos, A.; Tp, A.; Cournapeau, D. Scikit-Learn: Machine Learning in Python. *Mach. Learn. PYTHON*.

(6) Hunter, J. D. Matplotlib: A 2D Graphics Environment. *Comput. Sci. Eng.* **2007**, *9* (3), 90–95. https://doi.org/10.1109/MCSE.2007.55.

(7) Ryabchykov, O.; Bocklitz, T.; Ramoji, A.; Neugebauer, U.; Foerster, M.; Kroegel, C.; Bauer, M.; Kiehntopf, M.; Popp, J. Automatization of Spike Correction in Raman Spectra of Biological Samples. *Chemom. Intell. Lab. Syst.* **2016**, *155*, 1–6. https://doi.org/10.1016/j.chemolab.2016.03.024.

(8) Ryan, C. G.; Clayton, E.; Griffin, W. L.; Sie, S. H.; Cousens, D. R. SNIP, a Statistics-Sensitive Background Treatment for the Quantitative Analysis of PIXE Spectra in Geoscience Applications. *Nucl. Instrum. Methods Phys. Res. Sect. B Beam Interact. Mater. At.* **1988**, *34* (3), 396–402. https://doi.org/10.1016/0168-583X(88)90063-8.

(9) Erb, D. Pybaselines: A Python Library of Algorithms for the Baseline Correction of Experimental Data, 2025. https://doi.org/10.5281/ZENODO.16790579.

(10) Tolstik, E.; Lehnart, S. E.; Soeller, C.; Lorenz, K.; Sacconi, L. Cardiac Multiscale Bioimaging: From Nano- through Micro- to Mesoscales. *Trends Biotechnol.* **2024**, *42* (2), 212–227. https://doi.org/10.1016/j.tibtech.2023.08.007.

(11) Bergholt, M. S.; St-Pierre, J.-P.; Offeddu, G. S.; Parmar, P. A.; Albro, M. B.; Puetzer, J. L.; Oyen, M. L.; Stevens, M. M. Raman Spectroscopy Reveals New Insights into the Zonal Organization of Native and Tissue-Engineered Articular Cartilage. *ACS Cent. Sci.* **2016**, *2* (12), 885–895. https://doi.org/10.1021/acscentsci.6b00222.
(12) Movasaghi, Z.; Rehman, S.; Rehman, I. U. Raman Spectroscopy of Biological Tissues. *Appl. Spectrosc. Rev.* **2007**, *42* (5), 493–541. https://doi.org/10.1080/05704920701551530.
